# Downregulation of a CYP74 Rubber Particle Protein Increases Natural Rubber Production in *Parthenium argentatum*

**DOI:** 10.3389/fpls.2019.00760

**Published:** 2019-06-26

**Authors:** Dante F. Placido, Niu Dong, Chen Dong, Von Mark V. Cruz, David A. Dierig, Rebecca E. Cahoon, Byung-guk Kang, Trinh Huynh, Maureen Whalen, Grisel Ponciano, Colleen McMahan

**Affiliations:** ^1^Bioproducts Research Unit, Western Regional Research Center, Agricultural Research Service, United States Department of Agriculture, Albany, CA, United States; ^2^Guayule Research Farm, Section Manager Agricultural Operations, Bridgestone Americas, Inc., Eloy, AZ, United States; ^3^Department of Biochemistry, University of Nebraska–Lincoln, Lincoln, NE, United States; ^4^Monsanto Company, St. Louis, MO, United States

**Keywords:** allene oxide synthase, guayule, natural rubber, RNAi, jasmonic acid, salicylic acid

## Abstract

We report functional genomics studies of a CYP74 rubber particle protein from *Parthenium argentatum*, commonly called guayule. Previously identified as an allene oxide synthase (AOS), this CYP74 constitutes the most abundant protein found in guayule rubber particles. Transgenic guayule lines with *AOS* gene expression down-regulated by RNAi (*AOSi*) exhibited strong phenotypes that included agricultural traits conducive to enhancing rubber yield. *AOSi* lines had higher leaf and stem biomass, thicker stembark tissues, increased stem branching and improved net photosynthetic rate. Importantly, the rubber content was significantly increased in *AOSi* lines compared to the wild-type (WT), vector control and *AOS* overexpressing (*AOSoe*) lines, when grown in controlled environments both in tissue-culture media and in greenhouse/growth chambers. Rubber particles from *AOSi* plants consistently had less AOS particle-associated protein, and lower activity (for conversion of 13-HPOT to allene oxide). Yet plants with downregulated *AOS* showed higher rubber transferase enzyme activity. The increase in biomass in *AOSi* lines was associated with not only increases in the rate of photosynthesis and non-photochemical quenching (NPQ), in the cold, but also in the content of the phytohormone SA, along with a decrease in JA, GAs, and ABA. The increase in biosynthetic activity and rubber content could further result from the negative regulation of *AOS* expression by high levels of salicylic acid in *AOSi* lines and when introduced exogenously. It is apparent that AOS in guayule plays a pivotal role in rubber production and plant growth.

## Introduction

Natural rubber is a secondary metabolite synthesized by more than 2,500 plant species (Lewinsohn, 1991; Cornish, 2001; Konno, 2011), yet its function in plant physiology remains unclear. It has been proposed that natural rubber defends against pathogen or insect infestation, repairs tissue damage caused by mechanical wounding, and/or protects cell damage induced by environmental stress (Tangpakdee and Tanaka, 1998; Kim et al., 2003; Konno, 2011). Regardless of its biological role, natural rubber remains one of the most economically valuable agricultural products worldwide, and is indispensable for modern transportation, medicine, and national defense. *Hevea brasiliensis* (rubber tree) is the main source of natural rubber, but is limited geographically, susceptible to diseases such as leaf blight (Radziah and Chee, 1989; Nyaka-Ngobisa et al., 2013), and its latex causes allergic reactions in specific groups of individuals (Siler and Cornish, 1994). Alternative sources of natural rubber would assure adequate supplies, protect national security, and safeguard human health.

One plant known to be a promising source of natural rubber is guayule (*Parthenium argentatum* A. Gray), a desert shrub native to the southwestern United States and northern Mexico (Mooibroek and Cornish, 2000; van Beilen and Poirier, 2007). Most rubber synthesis in guayule occurs during the cold season when the shrub is semi-dormant (Downes and Tonnet, 1985; Gilliland and van Staden, 1986; Madhavan et al., 1989; Ji et al., 1993). Guayule synthesizes rubber within subcellular organelles (Archer and Audley, 1987) known as rubber particles (RPs), located in the stembark tissues (Gilliland et al., 1984; Macrae et al., 1986; Kajiura et al., 2018). These particles consist mostly of hydrophobic *cis*-1,4 polyisoprene (i.e., natural rubber) molecules partitioned inside a phospholipid and protein-containing membrane (Nawamawat et al., 2011; Sansatsadeekul et al., 2011). In guayule, the most abundant rubber particle protein is allene oxide synthase (AOS) (Backhaus et al., 1991; Whalen et al., 2013). AOS is well-known as the first committed enzyme in the jasmonic acid (JA) biosynthetic pathway (Harms et al., 1995; Wang et al., 1999; Wasternack and Song, 2017), but the function of high levels of AOS protein in rubber particles is unknown. Guayule AOS has been shown to be a monooxygenase, cytochrome P450, heme-binding protein (Backhaus et al., 1991; Pan et al., 1995; Brash, 2009) and a distinct member of the CYP74A enzyme family in that it does not require oxygen or NADPH, nor does it contain a chloroplast targeting sequence (Song and Brash, 1991; Song et al., 1993; Pan et al., 1995). Recent genome analysis estimates there are three *AOS* genes in diploid guayule (Valdes Franco et al., 2018). To date, however, only one guayule *AOS* gene has been cloned and characterized (*AOS1*). While in most plants AOS is present at relatively low levels, AOS CYP74A P450 in flax (*Linum usitatissimum*) accumulates relatively large quantities in seeds. The function of the high levels of AOS in flax seeds is likewise unknown.

Overexpression of flax seed *AOS* led to higher levels of JA in potato (*Solanum tuberosum*; Harms et al., 1995) and tobacco (*Nicotiana tabacum*; Wang et al., 1999). Similarly, increases in the JA content was observed when *Arabidopsis AOS* was overexpressed both in *Arabidopsis thaliana* and *N. tabacum* (Laudert et al., 2000) and rice *AOS* in *Oryza sativa* (Mei et al., 2006). Constitutive overexpression of *AOS* in the mutant *cea62* in rice also showed a dramatic increase in production of JA (Liu et al., 2012). In most cases wounding stress was a requirement for increased in JA level and substrate availability (Stenzel et al., 2003). On the other hand, down-regulation of *AOS* reduced or eliminated JA accumulation following wounding stress in *Arabidopsis* (Park et al., 2002) and further induced male sterility (Park et al., 2002; von Malek et al., 2002; Bae et al., 2010). These experiments are among those that documented *AOS* as the major control point in JA biosynthesis.

In this study, for the first time, a functional genomics approach was used to explore the role of *AOS* in guayule. We investigated the impact of *AOS* gene expression on guayule plant growth and development, phytohormone levels, and most importantly natural rubber production in plants grown in laboratory and greenhouse environments.

## Materials and Methods

### Plasmid Construction and Plant Transformation

The down-regulated and overexpressed guayule *AOS* gene in this study is the same gene reported in Backhaus et al. (1991), Pan et al. (1995, 1998), Chang et al. (2008), Li et al. (2008). The pND6 transformation vector was constructed from pPZP200 (Hajdukiewicz et al., 1994) with the potato *Ubiquitin 409* promoter (Rockhold et al., 2008) and the *Ubiquitin 1* sequence (Garbarino and Belknap, 1994) controlling β-*glucuronidase* (*GUSplus*) expression with the potato *409T* terminator on the 3′ end. The pPZP200 *Nos* promoter driving the *nptII* (Herrera-Estrella et al., 1983) gene for conferring kanamycin resistance with *Nos* terminator on the 3′ end was retained. The guayule *AOS* gene was amplified by PCR using cDNA as a template. Gene specific primers for amplification of *AOS* comprised the following sequences: 5′-cttaagaggtggtATGGACCCATCGTCTAAACCC-3′ and 5′-ggtccTCATATACTAGCTCTCTTCAGGG-3′. The resulting PCR products were subcloned into pGEM T Easy vector (Promega Corp., Madison, WI, United States) and sequenced. Subsequently, the *AOS* gene was excised using the *Afl*II and *Bam*HI restriction enzymes and inserted into pND6. Plasmid pND6::AOSoe (Supplementary Figure S1) was generated by replacing the *GUSplus* gene with full-length guayule *AOS* gene. Plasmid pND6::AOSi was constructed by replacing the *GUSplus* gene with an inverted *AOS* sequence repeat (Supplementary Figure S1). The repeat is a reverse complementary 313 bp sequence in its 5′ end and the forward 313 bp sequence in its 3′ end. A *BAR* gene (Christensen and Quail, 1996) of 552 bp was inserted into the middle of this inverted repeat. The plasmids pND6, pND6::AOSoe and pND6::AOSi were used to transform *Agrobacterium EHA101* competent cells (Hood et al., 1986). The transformed *Agrobacterium EHA101* strains were used to transform guayule cultivar G7-11 following Dong et al. (2013). The expression of the *GUSplus* reporter gene in the pND6 transformed guayule plants was confirmed by histochemical staining (Dong et al., 2006).

### Plant Germplasm and Growth Conditions

Wild-type (G7-11) and transgenic guayule lines were maintained in tissue culture as described in Dong et al. (2013). G7-11 is the source for publicly released USDA germplasm line AZ-2 (Dierig et al., 1989; Ray et al., 1999). It is apomictic, a tetraploid and a putative interspecific hybrid (Illut et al., 2017). This germplasm line is the most utilized line by industry as commercialization develops because of its vigor and rapid biomass accumulation. Our experiments included one WT, three *pND6* vector controls, four *AOSi* and four *AOSoe* independent lines. These lines are all considered highly genetically similar except for targeted genes and expression of affected genes since polyploid guayule reproduces apomictically. For daytime simulation, all plants were grown at a temperature of 25–27°C for 16 h; for night time simulation, cold-treated plants were grown at 10°C for 8 h in the dark, and room temperature (RT) plants remained at 25–27°C for 8 h in the dark. For phenotypic analysis (plant architecture measurements, gas exchange, chlorophyll fluorescence and rubber content), plantlets were carefully uprooted from the tissue culture medium and transplanted into a pot (6″ diameter × 4.25″ depth; ITML Horticulture, Canby, OR) with SS#1 F1P RSI 3.8 CFC CDN planting mix (Sun Gro Horticulture, McClellan Park, CA, United States). Plants were grown in the greenhouse for 1 month then transferred into Conviron E8 growth chambers (Conviron, Temecula, CA, United States) at 40–45% relative humidity, light intensity ∼150–200 μmol m^−2^ s^−2^ (Spectrum Technologies, Inc., Aurora, IL, United States) and subjected to cold treatment as described above for 2 more months, after which the plants were 5-months-old. At this point the growth chamber cold treatment showed increased rubber production for WT plants, as is well documented in field conditions. One month and 3 months cold treatment were tested but did not show significant differences in the natural rubber content. Cold treatment experiments were performed three separate times from August 2015 to March 2016. The design included 3–4 replications per line, placed randomly inside the growth chambers. Plants were watered and fertilized as needed. Gas exchange, chlorophyll fluorescence, plant height, number of stems, stem diameter and biomass were measured at the end of the 2-month exposure to cold stress. For rubber particle extractions, plants were grown in the greenhouse for 1 year, watered and fertilized as needed. The greenhouse conditions were the following: temperature, 25–27°C; relative humidity, 50–65%; and light intensity, ∼1100–1300 μmol⋅m^−2^⋅s^−2^ (Spectrum Technologies, Inc.).

### Genomic DNA Extraction and PCR Analysis

DNA was extracted using a Sigma GenElute Plant Genomic DNA Miniprep Kit (Sigma-Aldrich, Carlsbad, CA, United States). Approximately 150 mg leaf tissue was cut from the plants grown in tissue-culture, placed into 2 mL tubes and snap-frozen in liquid nitrogen. A bead was added to pulverize the tissues into a fine powder at a frequency of 30/s for 1 min using the mixer mill MM 400 tissue lyser (Verder Scientific, Inc., Newtown, PA, United States). PCR was carried out in 50 μL of a mixture containing GoTaq Green Master Mix (Promega Corp., Fitchburg, WI, United States), 200 ng guayule genomic DNA or 20 pg plasmid DNA, and 1 μM of vector and *AOS* specific primers 5′-CTTAAGAGGTGGTATGGACC-3′ and 5′-GGTTTCTTCCGGGTTCGAG-3′ for the *AOSoe* lines and 5′-ATGAGCCCAGAACGACGCCCGGCC-3′ and 5′-GATCTCGGTGACGGGCAGGACCGG-3′ for the *AOSi* lines. After heating the samples to 95°C for 2 min, the reaction proceeded with 35 cycles of 95°C for 30 s, 56°C to amplify the product ∼1.4 kb in the *AOSoe* lines (or 71°C for the product ∼0.5 kb in the *AOSi* lines) for 30 s and 72°C for 1 min. A final elongation step was carried out at 72°C for 5 min. PCR products were separated by electrophoresis on a 1% (w/v) agarose gel.

### RNA Extraction, cDNA Synthesis and qPCR

Leaf, stembark and root tissues were collected and snap-frozen in liquid nitrogen for total RNA extraction. RNA was extracted from ∼100 mg tissues using the TRIzol method (Ambion, Pittsburg, PA, United States), cleaned with RNeasy MiniElute kit (Qiagen Inc., Valencia, CA, United States) and DNase1 treated (Qiagen Inc., Valencia, CA, United States). RNA concentration was quantified with NanoDrop ND1000 (ThermoScientific, Wilmington, DE, United States).

The iScript cDNA synthesis kit (Bio-Rad, Hercules, CA, United States) was used to synthesize complementary DNA (cDNA) for real-time qPCR. Four μL of 5× iScript reaction mix, 1 μL of iScript reverse transcriptase, 1 μg of total RNA and nuclease-free water were used in a 20 μL reaction volume. The reaction mix was incubated in Eppendorf thermal cycler (Hauppauge, NY) with the following set-up: 5 min at 25°C for priming, 20 min at 46°C for reverse transcription, 1 min at 95°C for enzyme inactivation. For qPCR, 100 nM of forward and reverse primers, 7.5 μL of iQ SYBR Green Supermix Bio-Rad, Hercules, CA, United States), 2 μL of diluted cDNA (1:20) and nuclease free water were used in a 15 μL reaction volume. The 7500 Fast Real-Time PCR system (Applied Biosystem, Foster City, CA, United States) was use with the following thermal cycle: 95°C pre-incubation for 3 min; amplification for 40 cycles at 95°C for 15 s and 60°C for 30 s; the dissociation stage for 95°C for 15 s, 60°C for 1 min, and 95°C for 15 s. Each qPCR run was performed with three independent tissue samples, each sample having two technical replicates. The *18S* gene was used as an internal control with the following respective forward and reverse primer pair: 5′-CAACAAACCCCGACTTCTGG-3′ and 5′-CACCCGTCACCACCATAGTA-3′. The *AOS* gene specific forward and reverse primers used were: 5′-AACCCGGAAGAAACCAAACT-3′ and 5′-CGCAACCGACTGGAAATAAT-3′, respectively. These primers were designed such that the product to be amplified is external to the sequences used in down-regulating the *AOS* with the RNAi technology. The putative hydroperoxide lyase (HPL) gene specific forward and reverse primers used were: 5′-GGTTTCAAGGCTCAGAGACG-3′ and 5′-AACGCGCCTATCTCCAGTAA-3′, respectively. The putative divinyl ether synthase (*DES*) gene specific forward and reverse primers used were: 5′-GACTCTGGTCCGGTCAGCTA-3′ and 5′- CGGTTATCGGGACAATGGGT-3′, respectively. The melting curve data were collected for all samples and genes to ensure a single peak, indicating amplification of a specific region by a pair of primers. The relative expression values were calculated using the 2^−ΔΔCt^ method (Livak and Schmittgen, 2001). Expression of *AOS* gene was normalized to the expression of the endogenous reference *18S* gene and then to the expression of the WT plant.

### Gas Exchange and Chlorophyll Fluorescence Measurements

Simultaneous measurements of gas exchange and chlorophyll fluorescence were taken on leaf tissues using LI-COR 6400xt (LI-COR Biosciences, Lincoln, NE, United States). Measurements were conducted between 0900 and 1200 h. Plants were dark adapted 24 h prior to measuring chlorophyll fluorescence. The healthy and fully expanded middle leaf position was chosen because this position showed significant differences based on chlorophyll meter measurements (Spectrum Technologies Inc.; Supplementary Figure S4). The concentration of CO_2_ coming in from the CO_2_ mixer was held fixed at 400 μmol⋅s^−1^ with a constant flow rate of 500 μmol⋅s^−1^. The leaf (6 cm^2^) was clamped on the Li-COR cuvette under a leaf temperature of approximately 23°C, relative humidity between 60–65% and photosynthetic photon flux density (PPFD) artificially supplied by the red-blue light source at 1,000 μmol⋅m^−1^⋅s^−1^. Measurements were made once the leaf reached the stable net CO_2_ fixation rate. Three leaves were measured per plant and three to four plants per genotype were sampled.

### Natural Rubber Content

Fresh tissue from growth chamber plants (stems) and tissue-cultured plants (stems and leaves from 8 biological clones) was used for natural rubber content determinations. Tissues were harvested and lyophilized for 48 h. Tissues were then frozen in liquid nitrogen, ground to a fine power (30 Hz, 1 min) using a mixer mill MM 400 (Retsch, Haan, Germany), and stored at −80°C until analyzed. Approximately 300 mg of ground tissue was placed into an 11 mL ASE extraction cell (Dionex, Sunnyvale, CA, United States) with Ottawa sand as dispersant (Fisher Scientific, United States). A three-solvent sequential extraction method was applied (Pearson et al., 2013). Resin was extracted with acetone at room temperature, followed by a methanol extraction at room temperature, then rubber extracted with cyclohexane at 100°C (Fisher Sci., United States). Solvent containing analytes were evaporated using a TurboVap LV (Biotage, Charlotte, NC, United States) evaporator at 50°C with 15 psi N_2_. Rubber and resin dry weights were determined gravimetrically (w/w%).

### Rubber Molecular Weight

Gel permeation chromatography (GPC) was used to determine the natural rubber molecular weight. For tissue cultured plants, cyclohexane extractables collected from ASE were re-suspended in approximately 3 mL of tetrahydrofuran (THF; Fisher Sci., United States) with gentle, overnight shaking (Multi-Purpose Rotator, Thermo Sci., United States). For greenhouse plants, washed rubber particles were solubilized directly in THF. Solutions were syringe-filtered through a 1.6 μm glass microfiber Whatman grade GF/A filter (GE Healthcare Life Sciences, United States), then 50 μL injected onto a Hewlett Packard 1100 series HPLC and size exclusion separated (THF continuous phase, 1.0 mL/min) by two Agilent PL gel 10 μm Mixed-B columns in series at 35°C. The first peak elutes at ∼12 mins by refractive index (RID; Agilent 1260 Infinity, dn/dc = 0.129) and light scattering (DAWN Heleos-II, Wyatt Technology, Santa Barbara, CA, United States) detectors and represents the high molecular weight natural rubber. Measurements were repeated at least three times with three technical replicates performed for each genotype.

### Washed Rubber Particle Isolation

Rubber particles were extracted from WT, vector controls, and transgenic 1-year-old, greenhouse plants according to Cornish and Backhaus (1990). Briefly, ∼100 g of dirt-free stembark tissues were peeled off from each plant, ground twice, 1 min each, in a 4L blender containing 1L ice-cold extraction buffer. The resulting homogenate was filtered through eight layers of cheesecloth and distributed evenly in 250 mL centrifuge bottles. Centrifugation was performed in a swinging bucket rotor Sorvall RC5C 4°C centrifuge (DuPont, Wilmington, DE, United States) in a series of 2,000, 2,500, 3,500, 4,500, 5,500 and 7,000 rpm for 10 min. After each spin, the floating rubber particles were scooped with a stainless-steel spatula and transferred into 50 mL centrifuge tubes containing ∼25 mL ice-cold wash buffer and kept on ice. Purification by the aforementioned series of centrifugation speeds and subsequent collection into fresh, ice-cold wash buffer was performed two additional times. The rubber concentration (in μg⋅μL^−1^) was determined gravimetrically from three 50 μL aliquots of the final washed particles. Glycerol to a final concentration of 10% was added to the 3× washed rubber particles (WRPs) which were subsequently stored under liquid nitrogen until used.

### Rubber Particle Protein Extraction and Analysis

WRP proteins were extracted by adding 500 μL of WRPs into 500 μL extraction buffer (7 M urea, 2 M thiourea, 6.5 mM CHAPS, 1% Nonidet-40, 60 mM DTT, 5 mM EDTA). Samples were sonicated three times in 1 min intervals, resting on ice for 1 min after each sonication. To separate the solubilized proteins from rubber, samples were centrifuged at 14,000 *x g* at 4°C for 5 min, and aqueous fraction filtered with a Millex GV 0.22 μm filter (MilliporeSigma, Burlington, MA, United States). Proteins were further concentrated with Microcon-10 kDa centrifugal filter unit (MilliporeSigma, Burlington, MA, United States). Total protein concentration was estimated with Quick Start^TM^ Bradford Protein Assay (Bio-Rad, Hercules, CA, United States). After normalizing protein concentrations, the protein extracts were run on a 4–12% NuPAGE^®^ Bis-Tris pre-cast polyacrylamide gel under reducing conditions (Life Technologies, Carlsbad, CA, United States) and detected with Bio-Safe^TM^ Coomassie stain (Bio-Rad, Hercules, CA, United States). For western blot, proteins from the PAGE gel were transferred to a PVDF membrane, incubated for 45 min with 10 ng/mL anti-AOS-biotin conjugated monoclonal antibodies (Antibody Solution, Sunnyvale, CA, United States), following 30 min incubation with 2 ng/mL Streptavidin-Horse Radish Peroxidase conjugate (EMD Millipore, Temecula, CA, United States), and final chemiluminescent detection with SuperSignal^®^ Femto Maximum Sensitivity Substrate (Thermo Scientific, Waltham, MA, United States). Membrane was wrapped with plastic wrap and exposed to CL-XPosure^TM^ film (Thermo Scientific, Waltham, MA, United States) for 10–30 s. Recombinant AOS (Sigma-Aldrich, Saint Louis, MO, United States) was used as positive control. Protein molecular weight markers are PageRuler^TM^ Plus Prestained Protein Ladder (Thermo Scientific, Rockford, IL, United States).

### *In vitro* Assay of Rubber Synthesis

Enzymatically active WRPs were assayed for *in vitro* [1-^14^C]IPP incorporation in a Ultrafree^®^ MC-VV centrifugal filter unit (MilliporeSigma), in a 50 μL reaction volume containing ∼1 mg of RP, 20 μM farnesyl pyrophosphate (FPP) initiator, 1 mM unlabeled isopentenyl pyrophosphate (IPP), and 0.9 nmol (55 mCi⋅mmol^−1^) [1−^14^C]IPP in buffer (100 mM Tris–HCl, pH 7.5; 1.25 mM MgSO_4_, 5 mM dithiothreitol). The reactions, performed in triplicates, were incubated at 16°C for 3–4 h, stopped with addition of 40 mM ethylene diaminetetraacetic acid (EDTA), pH 8.0, and washed by centrifugation at 14,000 *x g* three times with water. The filter unit was inserted in a vial containing 2 mL ScintiVerse BD Cocktail (Fisher Sci., United States) and the amount of [^14^C]-IPP in each individual filter was quantified with LS 6500 scintillation counting instrument (Beckman Coulter, Brea, CA, United States).

### Rubber Particle Size

The size of extracted WRPs was determined using the Nano ZS ZEN 3600 (Malvern Instruments Ltd., United Kingdom) dynamic light scattering instrument. Briefly, the 3× WRPs extracted from 1-year-old greenhouse wild-type and transgenic guayule lines were filtered with cheesecloth. The sample volume (pH = 7.5) was adjusted to 1 mL with double distilled water. The liquid was transferred into the cuvette and inserted into the zetasizer instrument. The input refractive index was 1.51. The analyses were performed in triplicate. Data were analyzed using the average and standard deviation by student *t*-test. Significant differences were assessed relative to the wild-type and/or *pND6* controls, where values of *p <* 0.05 were considered statistically significant. Measurements were repeated at least three times with three technical replicates were performed for each genotype.

### Hormone Quantification

To determine the endogenous plant hormone content in the control and transgenic *AOS* plants using ESI-MS/MS, we followed the protocol by Pan et al. (2010) with modifications. Three biological plants with three technical replicates for each plant were used in this experiment. Briefly, leaves and stems were snapped-frozen and ground to powder with mortar and pestle. Solvent extraction solution and internal standards were added to ∼50 mg of pre-weighed tissues. ESI-MS/MS was performed as previously described (Luttgeharm et al., 2015) using a Shimadzu Prominence UPLC system, with ion detection by a QTRAP 4000 triple quadrupole mass spectrometer (Applied Biosystems) operated in negative mode and using previously published MRMs (Pan et al., 2010). Quantitation based on comparison of analyte to standard peak area was done using Multiquant 2.1 software (ABSciEX, Redwood City, CA, United States).

### Exogenous Salicylic Acid and Hydrogen Peroxide Treatment

Wild type G7-11 plants were grown on half-strength MS medium (PhytoTechnology Laboratories, Lenexa, KS, United States) in Magenta boxes (Caisson Labs, Smithfield, UT, United States) for 8 weeks with/out the addition of salicylic acid (SA) at the following concentration: 0, 10, and 100 μM diluted in 65% ethanol. For hydrogen peroxide, the following concentrations were tested: 0 nM, 0.1 nm and 1 nM diluted in distilled water. Leaf and stembark tissues were collected and snap-frozen in liquid nitrogen for RNA extraction and expression analysis by qPCR. For SA and hydrogen peroxide treatment of potting mix plants growing in growth chamber, 1-month old G7-11 plants were water with 50 mL solution containing 10 μM of SA and/or 0.1 nM of H_2_O_2_ directly into the potting mix three times per week for 8 weeks. Both SA and hydrogen peroxide treatment experiments were repeated at least three separate times with three biological replicates.

### AOS Enzyme Activity

Total proteins from 1 mL of WRPs extracted from stembark tissues of 1-year old greenhouse plants were solubilized in 0.5% CHAPS solution and sonicated three times at 1 min interval. Centrifugation at 14,000 *x g* at 4°C for 5 min followed thereafter. The clear solution (bottom layer) was aspirated from the rubber layer (top) into 1 mL syringe (Fisher Sci., United States) and passed through a Millex GV 0.22 μm filter (MilliporeSigma, Burlington, MA, United States). Total protein concentration was determined with Quick Start^TM^ Bradford Protein Assay (Bio-Rad, Hercules, CA, United States). For AOS activity, 400 ng of WRP total protein and 300 μM ethanolic solution of 13S-hydroperoxy-9Z,11E,15Z-octadecatrienoic acid (13S-HpOTrE; Cayman Chemical Co.; Ann Arbor, MI, United States) was added into 50 mM potassium phosphate buffer (pH = 7.0) to make a total volume of 200 μL. The 13S-HpOTrE was chosen because it is a precursor of JA (Song et al., 1993; Hughes et al., 2009). Reaction was carried out in specialized 96-well quartz microplate (Molecular Devices, San Jose, CA, United States) with absorbance measured at 235 nm in 3 s intervals over a 10-min period using SpectraMax M3 Multi-Mode Microplate Reader (Molecular Devices, San Jose, CA, United States). Vmax was determined based on the slope of optical density (OD) versus time in min, which was then normalized against WT to calculate the relative AOS activity. AOS activity was assayed in three biological replicates of each genotype.

### Hydrogen Peroxide Quantification

Hydrogen peroxide content was determined with optimized colorimetric determination using potassium iodide (Junglee et al., 2014). Eight-weeks-old guayule shoot (leaf and stem) were snap-frozen and ground to powder with mortar and pestle. One hundred fifty milligrams were homogenized in 1 mL of 0.25 mL of trichloroacetic acid (0.1% TCA; w/v), 0.5 mL potassium iodide (1 M) and 0.25 mL of potassium phosphate buffer (0.01 M; pH = 7) on ice for 10 mins with gentle shaking (Fisher Sci., United States). Homogenates were centrifuged in an Eppendorf 5403 at 15,000 *x g* for 15 min at 4°C. From each supernatant, an aliquot of 0.2 mL was aliquoted in a 96-well UV-microplate flat-round bottom (Greiner Bio-One Cat. #655801, Fisher Sci., United States) and incubated at RT (20–22°C) for 20 mins. Absorbance was measured for 1 min at 390 nm. The blank probe consisted of 0.1% TCA in the absence of the shoot extracts. H_2_O_2_ was quantified using a calibration curve of known H_2_O_2_ concentrations. Results are expressed as μmol H_2_O_2_ per gram of fresh weight.

### Peroxidase Activity Assay

The activity of peroxidase was assayed following the method described by Putter (1974) with a few minor alterations. Prior to sample collection, tissue-cultured WT and transgenic lines were treated at RT (25–27°C) and cold temperature (10°C) for 2 h in growth chamber with relative humidity of 50–65% and light intensity of ∼1100–1300 μmol⋅m^−2^⋅s^−2^ (Spectrum Technologies, Inc., United Kingdom). Approximately 0.5 g of shoot tissue was homogenized with 5 mL of phosphate buffer (0.1 M; pH 6.0) with mortar and pestle. Homogenate was transferred into a 15 mL tube followed by centrifugation in a Sorvall Instrument RC5C (DuPont) at 4,000 *x g* for 10 mins. A 50 μL aliquot was aspirated from the supernatant into a 96-flat bottom well (Greiner Bio-one Cat # 655101, Fisher Sci., United States). A 150 μL of reaction mixture consisting of phosphate buffer (1 M), guaiacol (0.23 M) and hydrogen peroxide (0.018 M) was added into the same 96 well plate. Absorbance was read at 470 nm in 15 s intervals for 2 mins using SpectraMax M3 Multi-Mode Microplate Reader (Molecular Devices, San Jose, CA, United States). Experiments were repeated at least three times with three technical replicates performed for each genotype. Activity per unit was calculated by 6 times the slope of the linear fit of A_470_ vs. time (min).

### Lipid Peroxidation Assay

Lipid peroxidation in tissue-cultured plants was determined by thiobarbituric acid (TBA) test (Heath and Parker, 1968). On ice, 0.1 mg of powdered shoot sample was added to 1 mL of trichloroacetic acid (TCA, 0.1%) in a 2 mL Eppendorf tube and mixed by vortexing. Samples were centrifuged for 10 mins at 13,000 rpm at 4°C. A 400 μL sample aliquot was mixed with 1 mL TBA (0.5%) and TCA (20%) mixture solution in a syringe-needle punctured cap 2 mL Eppendorf tube and incubated for 30 mins at 80°C. Tubes were immediately placed on ice for 5 mins followed by centrifugation at 13,500 rpm at 4°C for 5 mins. A 0.2 mL of the supernatant was aliquoted into the 96-well flat bottom Greiner plate (Greiner Bio-one Cat # 655101, Fisher Sci., United States) and absorption at 532 nm and 600 nm. The malondialdehyde (MDA) concentration was calculated using the following formula:

nmol MDA/ g FW = $\frac{\Delta A_{\text{corrected}}^{*}3.5*x*100}{\text{ε}*b*y}$; Δ*A*_corrected_ = A532–A600 corrected with ΔA of the blank; *b* = light path length (0.56 cm for 200 μl); ε = millimolar extinction coefficient (155 mM^−1^cm^−1^); 3.5 (dilution factor from 400 μl extract + 1 ml TBA/TCA solution); X (ml) TCA 0.1% used for extraction (1 ml); y (g) FW used for extraction; 1000 = conversion factor (nmol to μmol). Experiments were repeated at least three times with three technical replicates performed for each genotype.

### Statistical Analysis

Student’s *t*-test from MS Excel was used for all statistical tests.

### Accession Numbers

Sequence data from this article can be found in the GenBank data libraries under the following accession numbers: *GUSplus* (AF354045.1), potato *409T* terminator (HK352815.1) and guayule *AOS* (X78166.2).

## Results

### Generation and Confirmation of *AOS* Transgenic Lines

**FIGURE 1:**
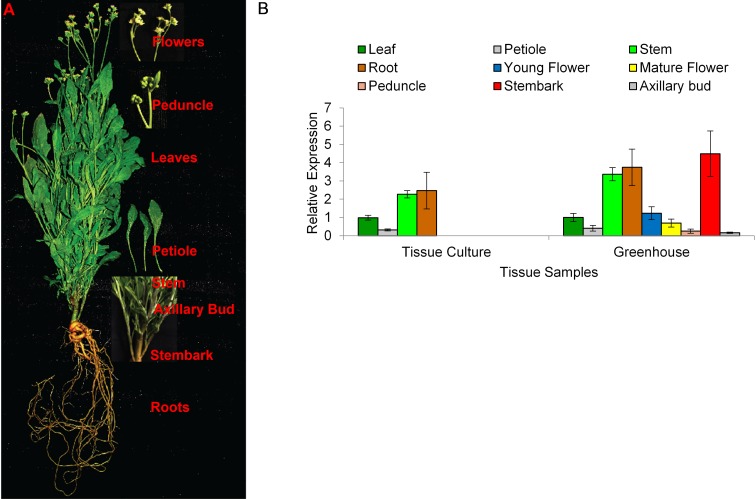
Endogenous *AOS* expression in guayule. **(A)** Guayule tissues analyzed. **(B)** *AOS* relative expression in WT G7-11 tissue-culture and greenhouse grown plant tissues. Expression levels for each tissue were compared to that of the corresponding leaf tissue, whether from tissue cultured or greenhouse grown plants, and normalized to the *18S* reference gene. Error bars represent SD from the average of three biological replicates.

While highly abundant in rubber particles, *AOS* can be detected in all tissues of guayule. The expression pattern of *AOS* in 8-week-old tissue-cultured and 2-month-old greenhouse grown plants revealed the highest level of *AOS* expression in stems and roots, and stembark tissues (Figure 1). Interestingly, natural rubber is also mainly found in the stembark tissues of mature plants and some in the roots and leaves (Curtis, 1947; Kuruvadi et al., 1997).

**FIGURE 2:**
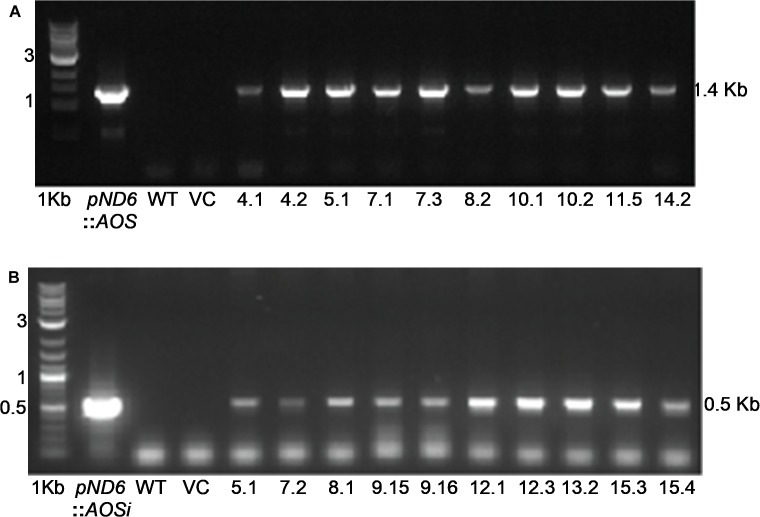
Genomic DNA PCR screening of **(A)** Overexpressing (*AOSoe*) and **(B)** downregulated (*AOSi*) transgenic guayule lines confirmed the presence of their corresponding transgenes. *pND6::AOSoe* and *pND6::AOSi*, transformation plasmid controls; WT, G7-11 wild-type control; VC, *pND6* vector control.

**FIGURE 3:**
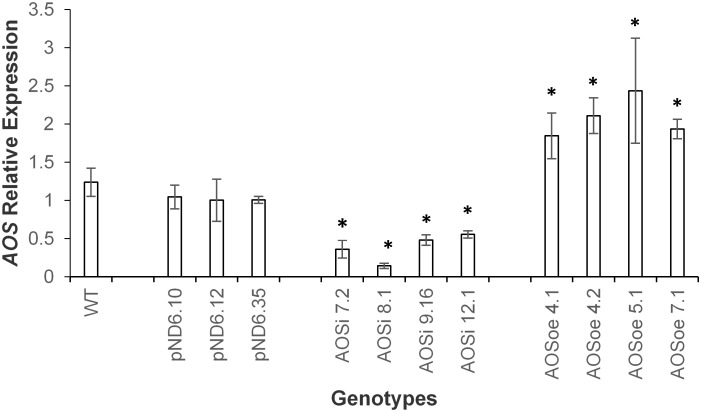
Expression analysis of *AOS* in guayule. WT, wild-type *G7-11*; *pND6*, vector control; *AOSi*, downregulated *AOS*; *AOSoe*, overexpressed *AOS*. Asterisks (^∗^) indicates significant difference in comparison to WT at *p* > 0.05. Total RNA from stembark of 5-month-old growth chamber-grown control and transgenic lines were template for qPCR. Expression levels were compared to WT and normalized to the *18S* reference gene. Average expression is from three biological replicates + SD.

**FIGURE 4:**
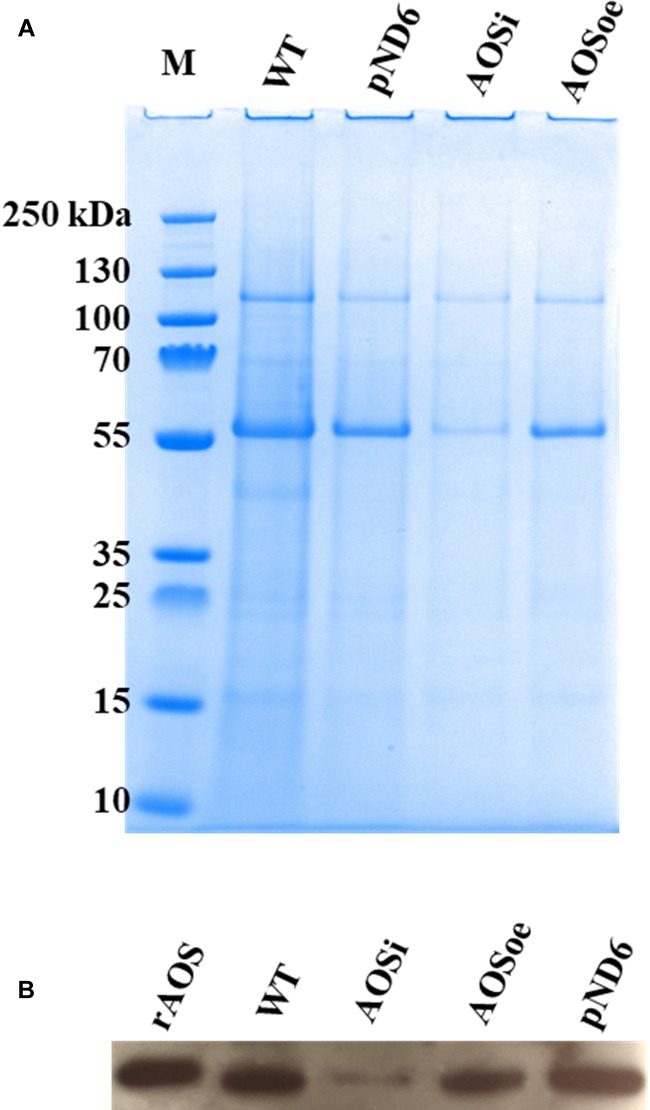
Washed rubber particle (WRP) proteins from guayule control plant and transgenic lines. Total proteins (3 μg) extracted from WRPs were separated by denaturing PAGE. **(A)** gel stained with Bio-Safe^TM^ Coomassie, and **(B)** western blot. WT, wild-type; pND6, vector control; AOSi, downregulated AOS; AOSoe, overexpressed AOS; rAOS, recombinant AOS protein (225 ng).

**FIGURE 5:**
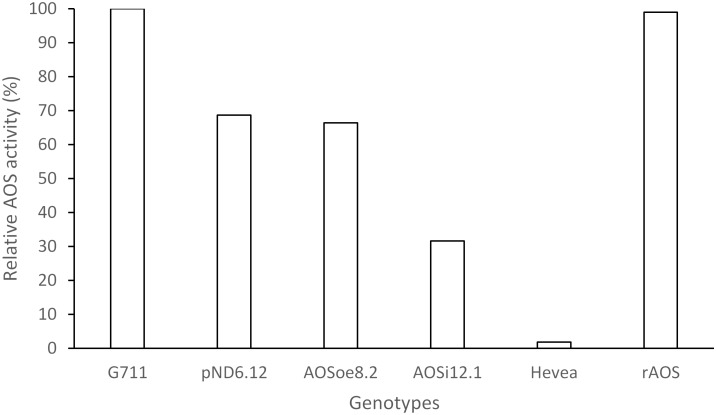
Relative AOS enzyme activity assay. WRP total protein extracts (400 ng) were assayed for AOS activity with 300 μM 13S-HpOTrE at 235 nm. Relative AOS activity was calculated by normalizing Vmax values against the WT. G7-11, wild-type control; *pND6*, vector control; *AOSi*, downregulated *AOS*; *AOSoe*, overexpressed *AOS*; *Hevea*, negative control; rAOS, commercial AOS recombinant protein, positive control.

To better understand the role of *AOS* in guayule, we generated transgenic plants in which *AOS* was overexpressed (*AOSoe*) or silenced (*AOSi*) by RNAi (Supplementary Figure S1). Four plants harboring the vector control (plant transformation plasmid with *GUSPlus* gene, *pND6*) were confirmed by GUS staining (Supplementary Figure S2). To confirm the presence of the *AOS* transgenes, genomic DNA from *AOS* transgenic lines was screened with PCR (Figure 2A,B). Ten each *AOSi* and *AOSoe* lines were found to be positively transformed. The expected impact of transgenes on *AOS* expression was confirmed with quantitative RT-PCR (qPCR) on total RNA from stembark tissues. *AOSi* lines had significantly lower levels of *AOS* expression and *AOSoe* lines had significantly higher levels of expression (Figure 3). Denaturing protein gel analysis (PAGE) of washed rubber particle total proteins from transgenic lines indicated reduced levels of AOS protein in *AOSi* lines (Figure 4A), confirmed by Western blot analysis (Figure 4B). Despite higher levels of *AOS* gene expression, *AOSoe* lines appear to not contain higher levels of AOS protein (Figure 4A,B). Moreover, relative AOS enzyme activity (13-HPOT conversion to allene oxide) was reduced as expected for the *AOSi* lines but not increased for the *AOSoe* lines compared to the *pND6* empty vector control (Figure 5). The discrepancy in the *AOS* transcript levels and protein levels and activity in the *AOSoe* lines indicates the absence of simple control of AOS protein levels.

Since rubber biosynthesis in guayule is highest in the cold (Goss et al., 1984; Ponciano et al., 2012), a series of experiments were conducted to evaluate the phenotype response of cold treatment on guayule plants varying in *AOS* expression. All plants were grown for 2 months at 25–27°C for 16 h in the light; RT plants were maintained at the same temperature for 8 h in the dark; cold-treated plants were grown at 10°C for 8 h in the dark.

### Downregulation of *AOS* Resulted in Larger Plants With Enhanced Photosynthesis

All *AOSi* lines were significantly taller (20 to 30%) and wider (28 to 30%) and had higher shoot (leaves and stems; 34 to 57%) and root biomass (50 to 61%; Table 1; Supplementary Figure S3) than WT, *pND6* and *AOSoe*. All *AOSi* lines also had more stems per plant than WT and *pND6* vector controls (Table 2). Additionally, the mature stembark tissues in *AOSi* lines had a thicker diameter (35 to 54%) in comparison to WT, *pND6* and *AOSoe* (Table 2). For all these phenotypes, cold treatment generally amplified the differences between *AOSi* lines and control lines.

**Table 1:** Phenotypes of 5-month-old guayule control and *AOS*-transgenic lines.

	Height (cm)		Shoot Biomass (g)		Root Biomass (g)	
Genotypes	RT	Cold	RT	Cold	RT	Cold
WT	21.0 ± 3.8	23.3 ± 0.2	6.3 ± 1.4	4.0 ± 0.7	1.2 ± 0.2	0.8 ± 0.1
*pND6.*10	25.5 ± 2.3	23.7 ± 2.8	5.7 ± 1.4	5.4 ± 0.6	1.0 ± 0.3	1.1 ± 0.1
*pND6*.12	25.0 ± 3.6	23.3 ± 2.1	6.1 ± 1.4	3.6 ± 1.0	0.9 ± 0.1	0.9 ± 0.2
*pND6.*35	22.7 ± 3.3	23.9 ± 1.0	5.7 ± 0.1	5.2 ± 0.7	1.2 ± 0.1	1.1 ± 0.05
*AOSi* 7.2	31.5 ± 0.4^∗∗^	31.3 ± 2.5^∗∗^	17.5 ± 5.5^∗∗^	15.8 ± 2.8^∗∗^	2.8 ± 0.9^∗∗^	2.5 ± 0.7^∗∗∗^
*AOSi* 8.1	33.8 + 2.4^∗∗^	30.8 ± 2.8^∗^	11.6 ± 2.7^∗∗^	9.7 ± 1.5^∗∗^	2.7 ± 0.9^∗∗^	2.7 ± 0.1^∗∗∗^
*AOSi* 9.16	34.0 + 1.7^∗∗^	31.3 ± 1.2^∗∗^	10.7 ± 0.9^∗∗^	11.2 ± 1.6^∗∗^	2.4 ± 0.8^∗∗^	2.3 ± 0.5^∗∗∗^
*AOSi* 12.1	30.2 ± 0.2^∗∗^	30.5 ± 3.7^∗^	9.5 ± 0.9^∗∗^	10.5 ± 2.5^∗∗^	2.4 ± 0.4^∗∗^	2.4 ± 0.2^∗∗∗^
*AOSoe* 4.1	24.3 ± 1.5	22.0 ± 2.6	4.0 ± 1.0	5.3 ± 0.8	0.9 ± 0.4	1.2 ± 0.3
*AOSoe* 4.2	25.8 ± 1.8	23.5 ± 1.3	4.6 ± 0.3	6.1 ± 0.4	1.1 ± 0.1	1.0 ± 0.04
*AOSoe* 5.1	25.3 ± 1.5	21.0 ± 1.0	7.3 ± 0.2	4.3 ± 1.0	1.0 ± 0.4	1.4 ± 0.05
*AOSoe* 7.1	24.3 ± 2.1	21.2 ± 2.0	5.2 ± 1.4	4.4 ± 0.8	0.9 ± 0.3	0.8 ± 0.2

*Values are average of three biological replicates ± SD. (^∗^), (^∗∗^), and (^∗∗∗^) indicate significant difference in comparison to WT at p > 0.05, 0.005, and 0.0005, respectively. Data are representative of three independent experiments.*

**Table 2:** Total branches and thickness of the primary stem of 5-month-old guayule control and *AOS*-transgenic lines.

Condition	RT				Cold				RT	Cold
			
Genotypes	Total branches per plant				Total branches per plant				Stembark thickness (mm)	
	#1	#2	#3	#4	#1	#2	#3	#4		
WT	1	2	4	–	3	4	4	–	0.8 ± 0.3	0.9 ± 0.01
*pND6*.10	1	3	4	0	3	4	5	–	1.2 ± 0.3	0.9 ± 0.3
*pND6*.12	2	3	4	–	2	3	3	–	0.7 ± 0.1	1.1 ± 0.03
*pND6.*35	1	2	3	3	1	1	4	1	1.1 ± 0.1	1.2 ± 0.1
*AOSi* 7.2	6	8	–	–	8	8	9	10	2.4 ± 0.7^∗^	1.7 ± 0.2
*AOSi* 8.1	7	8	9	10	8	8	10	11	2.0 ± 0.3^∗^	2.1 ± 0.2^∗∗^
*AOSi* 9.16	5	8	10	–	8	8	9	10	1.8 ± 0.01^∗^	1.7 ± 0.1^∗∗∗^
*AOSi* 12.1	4	5	6	–	5	6	6	6	1.6 ± 0.1^∗^	2.2 ± 0.2^∗∗∗^
*AOSoe* 4.1	3	3	3	–	4	4	5	–	1.0 ± 0.2	1.3 ± 0.1
*AOSoe* 4.2	1	3	3	–	1	3	3	5	1.1 ± 0.1	1.3 ± 0.04
*AOSoe* 5.1	2	3	4	5	3	5	5	–	1.0 ± 0.01	1.4 ± 0.0004
*AOSoe* 7.1	3	3	4	–	3	3	4	4	1.1 ± 0.2	0.9 ± 0.1

*Branch total was assessed by counting the number of branches per plant. Stembark thickness was determined by measuring the primary stem (bark and wood) minus the wood with a Mitutoyo digital caliper. Values of the branch thickness are the average of 3–4 biological replicates. (^∗^), (^∗∗^) and (^∗∗∗^) indicate significant difference in comparison to WT at p > 0.05, 0.005, and 0.0005, respectively. Data are representative of three independent experiments.*

The *AOSi* lines grown initially in the greenhouse and later in growth chambers were larger and had darker green leaves (Supplementary Figure S3) and higher chlorophyll levels (Supplementary Figure S4) than WT, *pND6* and *AOSoe*. Gas exchange rate measurements showed a higher (23 to 31%) photosynthetic rate (*Pn*) in *AOSi* lines compared to WT, *pND6* and *AOSoe* lines (Table 3). In addition, the *AOSi* lines showed higher stomatal conductance (*g*) and transpiration rate (*€*) as compared to all other genotypes (Table 3). Moreover, chlorophyll fluorescence measurements clearly showed efficiency of photosystem II (PSII) and electron transport rate (ETR) parameters are significantly higher in *AOSi* lines (Table 3) at both room and cold temperatures. Higher PSII and ETR would indicate more efficient light energy absorption and carbon assimilation in *AOSi* lines for growth and development as well as rubber synthesis compared to WT, *pND6*, and *AOSoe* genotypes. The non-photochemical quenching (NPQ) for *AOSi* lines was lower than controls under room temperature. However, under cold treatment, NPQ was higher in *AOSi* lines in comparison with WT, *pND6* and *AOSoe* genotypes, suggesting that *AOSi* lines have a more effective mechanism of protecting the PSII apparatus under cold stress.

**Table 3:** Gas exchange rate and chlorophyll fluorescence measured with Li-Cor of 5-month-old guayule control and *AOS*-transgenic lines.

Genotypes	*Pn* (μmol CO_2_ m^−2^ s^−1^)	g (μmol CO_2_ m^−2^ s^−1^)	€ (mol H_2_O m^−2^ s^−1^)	ΦPSII	ETR (μmol m^−2^ s^−1^)	NPQ

**RT**						
WT	5.75 ± 0.8	0.093 ± 0.03	2.33 ± 0.6	0.146 ± 0.015	115.73 ± 11.3	1.97 ± 0.2
*pND6.*10	6.29 ± 0.7	0.110 ± 0.03	2.74 ± 0.6	0.141 ± 0.015	111.1 ± 11.6	1.74 ± 0.1
*pND6.*12	6.20 ± 0.8	0.109 ± 0.02	2.66 ± 0.5	0.145 ± 0.015	114.4 ± 12.1	1.90 ± 0.2
*AOSi* 7.2	8.56 ± 0.6^∗∗∗^	0.147 ± 0.01^∗∗∗^	3.41 ± 0.3^∗∗∗^	0.196 ± 0.027^∗^	165.2 ± 10.7^∗∗∗^	1.33 ± 0.2^∗∗∗^
*AOSi* 8.1	8.40 ± 0.6^∗∗∗^	0.155 ± 0.02^∗∗∗^	3.67 ± 0.4^∗∗∗^	0.180 ± 0.008^∗^	141.4 ± 6.2^∗∗^	1.27 ± 0.2^∗∗∗^
*AOSi* 9.16	7.96 ± 0.5^∗∗∗^	0.162 ± 0.03^∗∗∗^	3.57 ± 0.5^∗∗∗^	0.186 ± 0.018^∗^	134.4 ± 3.3^∗∗^	1.19 ± 0.3^∗∗∗^
*AOSoe* 4.1	5.62 ± 0.9	0.110 ± 0.04	2.59 ± 0.7	0.133 ± 0.008	104.7 ± 6.7	1.92 ± 0.3
*AOSoe* 5.1	5.70 ± 0.8	0.110 ± 0.04	2.60 ± 0.7	0.133 ± 0.008	104.7 ± 6.7	1.95 ± 0.3
*AOSoe* 7.1	5.87 ± 0.9	0.113 ± 0.04	2.65 ± 0.7	0.137 ± 0.008	107.7 ± 6.3	2.07 ± 0.4

**Cold**						

WT	2.23 ± 0.5	0.054 ± 0.02	1.31 ± 0.4	0.070 ± 0.007	55.4 ± 5.4	1.58 ± 0.2
*pND6.*10	2.04 ± 0.4	0.057 ± 0.02	1.55 ± 0.3	0.064 ± 0.006	50.7 ± 4.9	1.56 ± 0.2
*pND6.*12	2.28 ± 0.4	0.065 ± 0.03	1.67 ± 0.7	0.066 ± 0.003	51.6 ± 2.7	1.51 ± 0.3
*AOSi* 7.2	4.14 ± 0.4^∗∗∗^	0.104 ± 0.02^∗∗∗^	2.54 ± 0.4^∗∗∗^	0.104 ± 0.011^∗∗∗^	81.0 ± 8.2^∗∗∗^	2.29 ± 0.3^∗∗^
*AOSi* 8.1	4.15 ± 0.4^∗∗∗^	0.112 ± 0.05^∗∗∗^	2.71 ± 0.8^∗∗∗^	0.102 ± 0.013^∗∗∗^	77.0 ± 4.5^∗∗∗^	2.33 ± 0.2^∗∗^
*AOSi* 9.16	4.14 ± 0.6^∗∗∗^	0.101 ± 0.03^∗∗∗^	2.60 ± 0.5^∗∗∗^	0.101 ± 0.009^∗∗∗^	79.4 ± 6.9^∗∗∗^	1.95 ± 0.1^∗∗^
*AOSoe* 4.1	2.27 ± 0.5	0.059 ± 0.02	1.51 ± 0.3	0.065 ± 0.011	60.4 ± 5.5	1.35 ± 0.3
*AOSoe* 5.1	2.97 ± 0.3	0.069 ± 0.01	1.81 ± 0.2	0.077 ± 0.007	58.5 ± 6.3	1.25 ± 0.2
*AOSoe* 7.1	2.45 ± 0.6	0.063 ± 0.01	1.93 ± 0.3	0.065 ± 0.010	50.1 ± 5.8	1.44 ± 0.2

*Pn, net photosynthetic rate; g, stomatal conductance; E, Transpiration rate; PSII, Efficiency of Photosystem II; ETR, Electron Transport Rate; NPQ, Non-photochemical quenching. Li-Cor readings are the average of three biological replicates ± SD. (^∗^), (^∗∗^) and (^∗∗∗^) indicate significant difference in comparison to WT at p > 0.05, 0.005, and 0.0005, respectively. Data are representative of three independent experiments.*

### Natural Rubber Biosynthesis Rate and Rubber Content Increased When *AOS* Was Downregulated

The natural rubber content in shoot tissues (stems and leaves) was increased in *AOSi* lines for both tissue cultured plants (in media) and growth-chamber grown plants (in potting mix). The *AOSi* lines produced more rubber (30 to 54%) than WT, *pND6* and *AOSoe* lines in the tissue-culture environment (Supplementary Table S1). Under cold treatment in growth chamber conditions, representing a microcosm of what guayule plants experience in the field, *AOSi* lines also exhibited higher rubber content in the shoots, with 16–21% more rubber in comparison with the WT (Table 4). Rubber also accumulated in the root tissues especially under cold treatment, where *AOSi* lines stored 18–38% higher rubber content compared with WT, *pND6* and *AOSoe* lines. In all cases *AOSoe* lines showed the same rubber content as control lines. Also, in all cases, terpene resins content showed no differences (data not shown).

**Table 4:** Rubber content of growth chamber-grown 5-month-old guayule control and *AOS*-transgenic lines by Accelerated Solvent Extraction (ASE).

	Average Rubber Content (%)			
	Shoot		Root	
Genotypes	RT	Cold	RT	Cold
WT 1	1.22 ± 0.09	1.13 ± 0.11	1.10 ± 0.04	0.95 ± 0.10
*pND6.*10	1.04 ± 0.18	1.31 ± 0.06	0.65 ± 0.07	0.72 ± 0.16
*pND6.*12	0.90 ± 0.06	1.14 ± 0.12	0.62 ± 0.08	0.82 ± 0.10
*pND6*.35	1.18 ± 0.08	1.27 ± 0.10	1.09 ± 0.06	0.94 ± 0.03
*AOSi* 7.2	1.49 ± 0.07^∗∗∗^	1.86 ± 0.11^∗∗∗^	0.56 ± 0.03	1.26 ± 0.09^∗∗^
*AOSi* 8.1	1.48 ± 0.05^∗∗∗^	1.91 ± 0.07^∗∗∗^	0.78 ± 0.14	1.19 ± 0.04^∗∗^
*AOSi* 9.16	1.46 ± 0.04^∗∗∗^	2.01 ± 0.08^∗∗∗^	0.66 ± 0.12	1.16 ± 0.02^∗∗^
*AOSi* 12.1	1.55 ± 0.07^∗^	1.80 ± 0.05^∗∗^	1.26 ± 0.04^∗∗∗^	1.52 ± 0.07^∗∗^
*AOSoe* 4.1	0.97 ± 0.26	1.13 ± 0.2	0.57 ± 0.05	0.79 ± 0.09
*AOSoe* 4.2	1.21 ± 0.10	1.30 ± 0.08	1.05 ± 0.10	0.94 ± 0.11
*AOSoe* 5.1	0.98 ± 0.30	1.04 ± 0.12	0.54 ± 0.11	0.62 ± 0.10
*AOSoe* 7.1	0.96 ± 0.30	1.15 ± 0.14	0.57 ± 0.06	0.55 ± 0.08

*Shoot tissue includes both leaves and stems. The percent rubber content (w/w%) is the average dry weight of three biological replicates ± SD. (^∗^), (^∗∗^) and (^∗∗∗^) indicate significant difference in comparison to WT at *p* > 0.05, 0.005, and 0.0005, respectively. Data are representative of three independent experiments.*

The molecular weight of the extracted rubber was analyzed to assess its quality. Under the tissue culture growing environment, when plants are very young (8 weeks old), *AOSi* lines showed significantly higher molecular weight rubber compared to all other genotypes (Supplementary Figure S5A). Natural rubber from all 1-year old plants in the greenhouse, however, displayed no differences in molecular weight (Supplementary Figure S5B).

**FIGURE 6:**
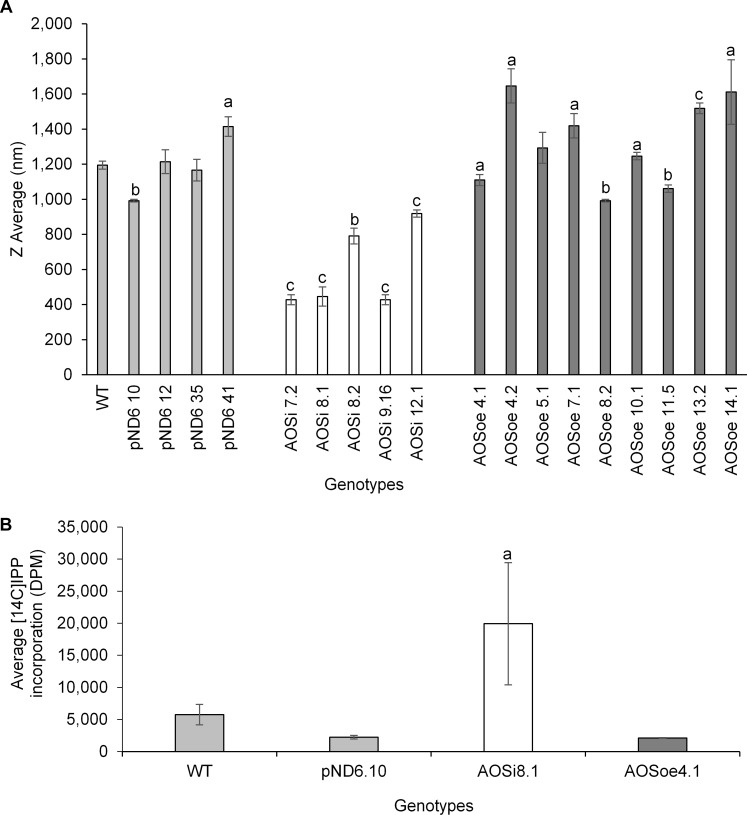
**(A)** WRP size determination by dynamic light scattering from 1-year old greenhouse-grown WT control and transgenic lines. Statistical significance analysis of the differences was performed with *t*-test with a, *p* < 0.05; b, *p* < 0.005; c, *p* < 0.0005 relative to the WT. **(B)** *In vitro* [^14^C]IPP incorporation assay. One mg of WRPs from 1-year old WT control and transgenic lines greenhouse plants was assayed in a final volume of 50 μL. Incorporation of labeled IPP was determined using a scintillation counter. Error bars represent SD from the average of three technical replicates. Data are representative of three independent experiments.

Washed rubber particles isolated from stembark tissues of greenhouse plants were analyzed by light scattering to assess particle size. WRPs from the *AOSi* lines had the smallest diameters compared to all other genotypes (Figure 6A). Importantly, the rubber transferase activity, quantified by ^14^C IPP incorporation into new polymer, was significantly enhanced in the *AOSi* WRPs (Figure 6B). Again, the *AOSoe* lines were equivalent to WT and *pND6* controls.

### *AOS* Downregulation Resulted in Less Jasmonic Acid, Abscisic Acid, Gibberellic Acid, 12-Oxo-Phytodienoic Acid and More Salicylic Acid

AOS is the first enzyme committed to the production of JA, which was reduced as expected in *AOSi* lines (Table 5). Hormone profile analysis of tissue-culture grown lines revealed the content (ng/gfw) of JA and its precursor, 12-oxo-phytodienoic acid (OPDA), were significantly reduced to half (or less, down 10-fold) of that found in control lines. Abscisic acid (ABA) and gibberellic acid (GA), were diminished in the *AOSi* lines compared to WT, *pND6* and *AOSoe* lines, to a lesser but still significant extent (Table 5). Conversely, the SA content was higher in the *AOSi* lines than in the rest of the lines by about 2-fold. Reducing expression of *AOSi* in guayule was associated with reduced levels of JA and other hormones as well, but higher levels of SA.

**Table 5:** Liquid chromatography-tandem spectrometry hormone analysis of stem and leaf tissue from 8-week-old tissue-cultured guayule control and *AOS*-transgenic lines.

	Content (ng/gfw)						
Genotypes	Jasmonic Acid	Salicylic Acid	Abscisic Acid	Gibberellin A_20_	Gibberellin A_1_	Gibberellin A_3_	12-oxo-phytodienoic acid
WT	5.36 + 1.2	5.50 + 0.8	11.01 + 1.9	15.95 + 0.7	9.95 + 0.07	3.52 + 0.2	2.96 + 0.7
*pND6.12*	1.57 + 0.1	4.89 + 0.6	7.05 + 0.8	14.11 + 1.2	12.39 + 2.2	2.19 + 0.01	5.44 + 0.3
*pND6.33*	4.76 + 1.0	5.04 + 0.1	7.24 + 0.3	13.86 + 2.6	12.70 + 2.3	1.79 + 0.09	3.60 + 0.8
*pND6.35*	1.96 + 0.4	5.6 + 0.5	7.10 + 0.6	14.41 + 1.2	14.65 + 0.2	2.16 + 0.3	2.17 + 0.3
*AOSi 7.2*	0.57 + 0.1_∗∗_	9.51 + 0.5^∗^	4.71 + 0.6^∗^	9.63 + 1.2^∗^	5.13 + 0.8^∗^	0.86 + 0.3^∗^	0.76 + 0.2^∗^
*AOSi 9.16*	0.57 + 0.01_∗∗_	7.65 + 0.2^∗^	2.96 + 0.3^∗^	8.85 + 2.1^∗^	7.81 + 0.2_∗∗_	0.80 + 0.3^∗^	0.39 + 0.07^∗^
*AOSi 12.1*	0.68 + 0.05_∗∗_	9.65 + 1.1^∗^	3.64 + 0.5^∗^	10.59 + 0.2^∗^	6.75 + 1.1^∗^	1.32 + 0.07^∗^	0.81 + 0.14^∗^
*AOSoe 4.1*	1.48 + 0.2	4.03 + 0.7	9.13 + 1.71	15.49 + 0.6	10.78 + 0.1	1.93 + 0.06	2.33 + 0.1
*AOSoe 4.2*	3.25 + 0.2	4.76 + 0.6	13.9 + 1.3	no data	14.63 + 2.7	1.80 + 0.1	2.84 + 0.8
*AOSoe 7.1*	1.41 + 0.3	5.50 + 0.02	8.84 + 0.3	13.64 + 1.2	13.96 + 0.01	1.84 + 0.1	2.68 + 0.7

*Values represent the average ± standard deviation of three biological replicates. (^∗^) and (_∗∗_) indicates significant difference in comparison to G7-11 at *p* > 0.05 and 0.005, respectively.*

### Exogenous Treatment With Salicylic Acid Enhanced Rubber Content

SA (or its analogs) is known to have an inhibitory effect on *AOS* transcription (Harms et al., 1995) and activity (Pan et al., 1998; Norton et al., 2007). When SA was applied to guayule WT plants grown in tissue culture, the expression of *AOS* decreased, and the amount of rubber increased, in a dose-dependent manner (Supplementary Figures S6A,B). Under growth chamber conditions, SA treatment in the potting mix reduced *AOS* expression in guayule stems (Table 6). Rubber content increased, and plant architecture was impacted (increased height and stem diameter; Table 6) with SA potting mix treatment relative to mock or water treated WT plants. We generally observed that, in guayule, exogenous SA treatment resulted in similar phenotypes to *AOSi* downregulation.

**Table 6:** Exogenous treatments with SA and H_2_O_2_. *AOS* relative expression in the stem, rubber content and phenotypes of 5-month-old WT guayule plants.

RT						
Treatment	*AOS* relative expression	Rubber content	Height (cm)	Biomass (g)	Stembark thickness (mm)	Total branches
Mock	1.65 ± 0.57	0.63 ± 0.12	18.9 ± 2.3	9.0 ± 1.9	1.919 ± 0.134	4.0 ± 1.6
SA	0.44 ± 0.07^a^	1.08 ± 0.17^a^	25.0 ± 2.4^a^	10.4 ± 2.4	3.071 ± 0.422^b^	4.3 ± 0.5
H_ 2_O_2_	0.24 ± 0.12^a^	0.91 ± 0.04^a^	19.3 ± 2.8	10.1 ± 3.6	2.6393 ± 0.238^a^	4.8 ± 1.3
SA + H_2_O_2_	1.39 ± 0.35	0.61 ± 0.14	21.0 ± 2.7	10.1 ± 3.5	2.280 ± 0.457	5.0 ± 0.8

**Cold**						
Mock	1.49 ± 0.39	0.60 ± 0.08	32.4 ± 1.2^d^	14.6 ± 1.4^d^	1.785 ± 0.569	4.5 ± 1.0
SA	0.47 ± 0.08^a^	1.04 ± 0.07^c^	29.3 ± 2.2^a,c^	15.9 ± 1.8^c^	2.195 ± 0.134^c^	5.5 ± 1.0
H_2_O_2_	0.61 ± 0.12^a^	0.88 ± 0.06^b^	33.8 ± 1.8^c^	15.1 ± 1.7^c^	1.763 ± 0.322^d^	4.0 ± 0.8
SA + H_2_O_2_	0.11 ± 0.04^a,d^	0.91 ± 0.03^b,d^	29.8 ± 1.8^a,d^	16.0 ± 0.6^c^	2.180 ± 0.166	6.5 ± 0.6^a,c^

*Values represent the average ± standard deviation of three biological replicates. a and b are significantly different within treatments; c and d are significantly different between treatments. Data are representative of three independent experiments.*

### Hydrogen Peroxide Levels and Peroxidase Activity Are Impacted by *AOS* Downregulation

**FIGURE 7:**
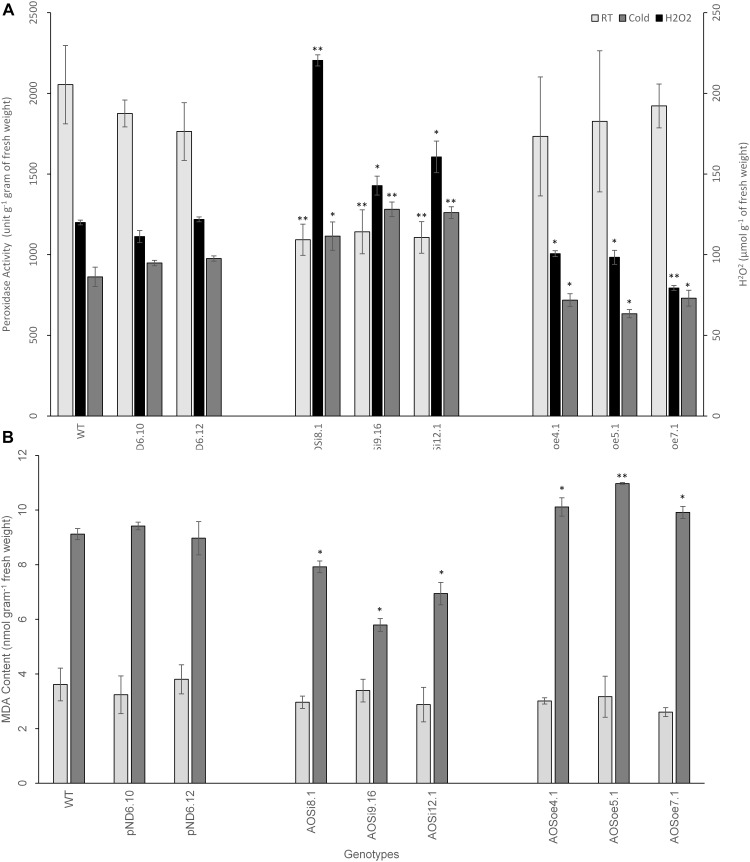
Spectrophotometric measurement from 8-week-old tissue culture-grown guayule control and transgenic lines for **(A)** H_2_O_2_ level and peroxidase activity and **(B)** lipid peroxidation as measured by MDA content *in planta*. Average values are from three biological replicates ± SD. ^∗^ and ^∗∗^ indicate significant difference at *p* > 0.05 and *p* > 0.005, respectively, in comparison to the WT. Data are representative of three independent experiments.

SA is known to influence hydrogen peroxide (H_2_O_2_), in either a self-amplifying or antagonistic manner (Quan et al., 2008). We tested the impact of *AOS* downregulation on peroxide level and peroxidase activity in tissues. Hydrogen peroxide levels measured *in planta* were higher for *AOSi* lines than for controls, under unstressed conditions (Figure 7A), suggesting enhancement between H_2_O_2_ and SA (and antagonistic to JA). Extracts from the *AOSi* lines had significantly lower peroxidase activity than WT, *pND6* and *AOSoe* lines in a RT growth environment, consistent with the higher H_2_O_2_ level (Figure 7A). When plants were cold-treated, the WT, *pND6* and *AOSoe* genotypes showed lower peroxidase activity compared to the RT condition. However, the *AOSi* plants showed no change in peroxidase activity with cold treatment (Figure 7A).

### Exogenous Treatment With Hydrogen Peroxide Also Enhanced Rubber Content

Exogenous treatment of WT plants with H_2_O_2_ reduced *AOS* expression (Table 6), and increased rubber content relative to untreated plants both in RT and cold treatments (Table 6). Under room temperature, thickness of the primary stembark was significantly larger. However, thickness of the primary stembark was considerably smaller in cold treated plants (Table 6). Cold significantly increased the height and biomass of all plants compared to RT (Table 6). Other plant growth parameters did not show differences. The addition of both SA and H_2_O_2_ together did not enhance the rubber content under room temperature; *AOS* expression was reduced only under cold treatment with a concomitant increase in rubber content (Table 6).

### Lipid Peroxidation Is Reduced Under Cold Stress for Downregulated Lines

Previous studies have suggested that plants produce natural rubber in order to maintain the integrity of the cellular membrane and keep protein-membrane interaction intact upon and/or during stress (Tangpakdee and Tanaka, 1998; Kim et al., 2003). We tested whether the differences in peroxidase activity may have resulted in cellular damage from oxidation. A lipid peroxidation assay was used to estimate the integrity of the cellular membrane for the tissue culture-grown WT and transgenic lines. This method measured the amount of the product of lipid peroxidation, malondialdehyde (MDA). High MDA levels generally indicate internal damage to the cells due to reactive oxygen species such as H_2_O_2_. No differences were observed in MDA content between genotypes for guayule plant shoot tissues grown in RT (Figure 7B). However, when cold treatment was imposed, the MDA level increased significantly, suggesting stress-induced cellular damage. Further, the MDA level was slightly lower for the *AOSi* lines and higher for the *AOSoe* lines in comparison to the WT and *pND6* controls. Therefore, under cold treatment, the higher peroxidase activity in the *AOSi* lines (Figure 7A) may have mitigated oxidative stress thereby reducing cell wall damage in *AOSi* lines (Figure 7B). Sundar et al. (2004) reported that protective antioxidant metabolism, including peroxidase activity, increased to mitigate such oxidative stress in guayule exposed to low temperatures.

## Discussion

A functional genomics approach was used to dissect the role of *AOS* in guayule. AOS is a member of the CYP74A enzyme family that metabolizes 13-hydroperoxylinolenic acid (13-HPOT) into allene oxide leading to JA synthesis (Song et al., 1993; Hughes et al., 2009). Levels of phytohormones and plant phenotypes associated with rubber production and growth were investigated in transgenic lines in which *AOS* was downregulated (*AOSi*) or overexpressed (*AOSoe*). Surprisingly, given its prevalence in the rubber-producing sub-cellular rubber particles, down-regulation of *AOS* in guayule (*AOSi*) resulted in higher natural rubber content (weight/weight %) compared to the WT, *pND6* controls and overexpressed *AOS*-transformed plants. Interestingly, the physically larger *AOSi* guayule lines maintained a higher rate of photosynthesis conducive to increased carbon flux and rubber biosynthesis. Salvucci et al. (2010) linked guayule’s carbon fixation rate to higher rubber content (but not higher biomass) in greenhouse plants grown in winter-like conditions. Here, increased photosynthetic activity led to both higher biomass and higher rubber content in *AOSi* lines. Moreover, *AOSi* lines were associated with significant architectural changes which could contribute to enhanced natural rubber yield. Hormonal balances were disrupted in the *AOSi* lines, with changes that ultimately led to increased branching and stem thickness, for instance. Phytohormone interaction networks are complex, and it is clear that additional studies would be required to directly tease out cause and effect of manipulating *AOS* expression in guayule. Importantly, there were no discernible phenotypes associated with *AOS* overexpression, regardless of the scale used. The lack of an *AOSoe* phenotype suggests active control of protein levels, for example at the translational level or through a degradation mechanism. Evidence for transcriptional and post-transcriptional regulation in the JA signaling pathway has been previously reported in *Hevea* (Pirrello et al., 2014).

While the evolutionary function of rubber in plants is unknown, it has been suggested that natural rubber production serves as a protective mechanism against abiotic and biotic stresses (Tangpakdee and Tanaka, 1998; Kim et al., 2003). Our studies suggest that JA and SA levels may play a role in translation of stress signals relevant to natural rubber biosynthesis in guayule. Young guayule plants with lower endogenous JA due to down-regulation of *AOS* had consistently higher rubber content. In *Hevea*, exogenous application of JA (and its precursor, linolenic acid) increased the number of primary laticifers in young *Hevea* plants (Hao and Wu, 2000), which is related to higher rubber yield (Yu et al., 2008; Tan et al., 2017).

This generally contrasting result could be explained by a multitude of possibilities. It is not unexpected that there would be many physiological differences between the cells in stembark tissues (Kajiura et al., 2018) where rubber is synthesized in guayule, and laticifer cells, where it is synthesized in *Hevea* and *Taraxacum kok-saghz* (another rubber producing plant). In *T. kok-saghz*, treatment with methyl JA (MeJA) upregulated the expression of *AOS* and other JA associated genes (Cao et al., 2017). These results indicate a feedback loop mechanism exists connecting JA and *AOS* in *T. koksaghyz*. Conversely, in *Hevea*, treatment with JA had no effect on AOS enzyme activity (Norton et al., 2007).

AOS is well-known for its role in the biosynthesis of JA, but may have other functions as well (Pan et al., 1998). Furthermore, we cannot rule out unintentional silencing of other CYP74 family members such as three putative *AOS-like* sequences identified in a guayule stem RNASeq database (Supplementary Figure S7). Two of those sequences are likely isoforms of AOS as they share 87 and 79% amino acid identity with AOS (AOSL2 and AOSL3, respectively, in Supplementary Figure S7. This is not surprising as the recent guayule draft genome predicts three *AOS* genes (Valdes Franco et al., 2018). Although the RNAi construct and the potential target region of *AOSL2* and *-3* are not perfectly matched, it is possible they could be silenced. Silencing of *AOSL2* and *-3* could certainly contribute to some of the phenotypes observed in the *AOSi* lines. However, since none of the deduced proteins of *AOSL2* and *-3* appear to associate with the RP (Kajiura et al., 2018), silencing of these sequences would not directly affect the rubber biosynthetic complex. The third sequence (*AOSL4*) encodes a putative protein of 147 amino acids (a little over half the size of AOS) with 99% identity to AOS C-terminus. The RNAi construct matches this novel protein 100% and thus is likely silenced. Rubber particle proteomic analysis, however, did not identify this novel protein (Kajiura et al., 2018) and therefore its impact on rubber synthesis if any, remains unknown.

Other potential RNAi off-targets are DES and HPL, both CYP74 enzymes. Like AOS, DES, and HPL are key enzymes of plant oxylipin metabolism (Hughes et al., 2009) and their silencing would disrupt oxylipin signaling other than JA, with potential consequences in plant development. For example, colnelenic acid, a product of DES, affected root development in *A. thaliana* seedlings (Hughes et al., 2009). Putative guayule *DES* and *HPL* transcript sequences were cloned by PCR and sequenced. Expression analysis of these two putative genes showed down-regulation of *DES* but not *HPL* (Supplementary Figure S7) in the *AOSi* lines. It is possible therefore that some of the phenotypes observed in the *AOSi* lines are in part due to down-regulation of *DES*.

Down-regulating *AOS* in guayule not only decreased the amount of JA *in planta*, but also impacted other endogenous plant hormones suggesting a broad disruption in signaling. Importantly, SA levels were higher in the *AOSi* lines compared to WT, *pND6* and *AOSoe* genotypes, suggesting that *AOS* directly or indirectly plays a role in suppressing SA production. The indirect route may be mediated by JA, as the two hormones are known to have an antagonistic relationship. On the other side of the interaction, SA treatment of guayule resulted in downregulation of *AOS* expression (Table 6). Most notably, the *AOS* expression, physical phenotypes and rubber content (Figure 3 and Tables 1, 2, 4) of the SA-treated plants (Table 6) showed similar phenotypes to the *AOSi* lines.

Salicylic acid is known to affect plant growth and development, photosynthesis, and stress response. Since JA is conversely known to inhibit photosynthesis (Qi et al., 2015; Zhu et al., 2015), the enhanced photosynthetic rate could be due to lower JA, higher SA, or both. In *AOSi* lines; higher levels of SA not only lower barriers to photosynthesis but could also enhance the efficiency of anti-oxidative response during cold stress. Further evidence was observed under cold treatment when NPQ was higher in *AOSi* lines in comparison with WT, *pND6* and *AOSoe* suggesting that *AOSi* lines have a better PSII system protection in response to stressful environment. This result conforms with that of Turan et al. (2014), where guayule plants’ NPQ increased systematically with light exposure. They suggested that the higher NPQ served to alleviate the occurrence of photo-oxidative damage and thereby acclimate the plants to high light stress. Therefore, increases in SA, by enhancing photosynthesis, along with a more efficient cold stress response (i.e., higher NPQ) in guayule *AOSi* lines, result in more carbon supply for rubber synthesis and growth. These observations corroborate earlier reports by Allen et al. (1987) and Sundar and Reddy (2000) regarding the contribution of higher photosynthetic rates to biomass and rubber formation in guayule under cold conditions.

SA’s protective capacity in response to environmental stress in plants, sometimes functions antagonistically to JA (Pieterse et al., 2012), thus allowing the plant to fine tune its stress response and efficiently utilize its resources (Walters and Heil, 2007). The observed higher SA levels in *AOSi* guayule plants may have been a consequence of the lowered JA synthesis, and induced downstream stress responses. Rubber biosynthesis, usually initiated by cold stress, may instead respond to SA signaling in *AOSi* plants. Moreover, exogenous addition of SA or H_2_O_2_ increased rubber content in guayule, possibly as an induced stress response. Guayule AOS is competitively inhibited by SA (Pan et al., 1998) so it is not surprising that addition of SA and downregulation of *AOS* produced similar phenotypes. It is tempting to speculate that SA used as a foliar spray on guayule would circumvent the need for seasonal cold periods to maximize rubber production.

Downregulation of *AOS* reduced the plant tissues’ content of JA, it’s precursor, OPDA, several GAs, and ABA compared to the controls and overexpressed lines, probably due to hormonal crosstalk (Hou et al., 2013; Wasternack and Hause, 2013; Song et al., 2014). Stem morphology phenotypes have been associated with lower concentration of GA, using mutants or inhibitors, resulting in cortical cell expansion in pea plants (*Pisum sativum*; Tanimoto, 2012) and production of thicker stems in tobacco (Biemelt et al., 2004), consistent with thicker stems found for guayule with lower GA. The guayule *AOSi* lines also exhibited more branching, along with lower ABA content compared to the controls and *AOSoe* lines. Disruption in ABA signaling has been shown to negatively regulate auxillary bud growth (Yao and Finlayson, 2015), including in transgenic poplar (*Populus* × *canescens*), where reduced sensitivity or lower detectability toward ABA resulted in more lateral bud growth (Arend et al., 2009).

Lastly, OPDA, precursor to JA, has been reported to have a role, independent of JA, in stomatal closure in response to drought in *Arabidopsis*, tomato and *Brassica napus* (Savchenko et al., 2014). The Savchenko et al. (2014) study correlated higher OPDA, independent of and in concert with ABA, with reduced stomatal aperture. Therefore our observed physiological phenotypes showing low OPDA, higher stomatal conductance, and improved photosynthetic rate in the *AOSi* lines is a pertinent and significant finding. Overall, by disrupting hormonal balances in the *AOSi* lines, a guayule plant was developed that exhibited higher biomass accumulation from higher shoot and root biomass, increased stem branching, thicker stembark tissues and enhanced photosynthetic rate, all advantageous traits for increased natural rubber production.

A remaining question is why do guayule plants maintain high levels of AOS on the surface of rubber particles? The significant decrease in peroxidase activity found in the *AOSi* lines provides evidence that guayule rubber particle AOS may have an alternative or additional function in addition to or instead of JA biosynthesis. Pan et al. (1998) found recombinant guayule AOS to have a higher affinity for 15S-HPETE (an animal lipid derivative) than for 13S-HPODE or 13S-HPOTE (the usual plant substrates). Another AOS from flaxseed (Song et al., 1993) that is highly homologous to AOS from guayule, except for the chloroplast localization signal sequence (Pan et al., 1998), can also metabolize 15S-HPETE, suggesting alternative, but still unknown, functions in the two plant species.

The *AOSi* lines’ rubber particles showed reduced particle size as well as higher rubber transferase activity. In *Hevea*, smaller rubber particles (SRP) have higher transferase activity compared to the larger rubber particles (LRP; Ohya et al., 2000; Rojruthai et al., 2010; Schmidt et al., 2010; Yamashita et al., 2017); and our results provide the first evidence that this may be the case in guayule. Dai et al. (2017) propose a role for the two most abundant proteins in *Hevea* rubber particles (rubber elongation factor, REF, and small rubber particle protein, SRPP): as rubber is synthesized and the rubber particles grow in size, REF and SRPP associate to maintain the needed structure to stabilize the particle as the volume increases. It is possible then that in guayule less AOS on the particle surface may prevent enlargement and consequently retaining a higher rubber transferase activity in *AOSi* lines, leading to a more active form of the transferase complex. Hughes et al. (2006) proposed that differences in *Arabidopsis* CYP74 enzyme activity and substrate specificity are related to the extent of membrane association, even to the point of solubilization. They further pointed out that during pest or pathogen attack, AOS may be released from its membrane-bound state. The possibility that release of AOS from guayule rubber particles, for example, under cold stress, contributes to increased rubber biosynthesis, suggests a new view of the role of the abundant CYP74 rubber particle protein in guayule. This opens several possible avenues to pursue further, including assessment of conformational changes of the rubber transferase complex, and dissecting the role of SA in transducing the cold-induced increase in rubber production and enhancement in biomass. In conclusion, downregulation of *AOS* in guayule resulted in a plant with disruption of endogenous hormones, and higher photosynthetic rate, biomass, and natural rubber content. Furthermore, SA was found to be a mediating factor in the regulation of *AOS* expression.

## Data Availability

The datasets generated for this study can be found in NCBI, Sequence data from this article can be found in the GenBank data libraries under the following accession numbers: GUSplus (AF354045.1), potato 409T terminator (HK352815.1) and guayule AOS (X78166.2).

## Author Contributions

DP, ND, MW, and CM conceived and performed the original research plans and supervised the experiments. DP, GP, MW, and CM wrote the manuscript. GP performed rubber transferase assay, PAGE and western blot analysis. DP and CD performed the AOS enzyme activity and hydrogen peroxide assays, rubber and latex extractions and washed rubber particle analysis. VC and DD provided technical assistance and complemented the writing. RC performed the hormone analysis. ND prepared transformation vectors. ND, B-gK, and TH performed the guayule plant transformation and maintenance of the tissue-cultured plants.

## Disclaimer

Mention of trade names or commercial products is solely for the purpose of providing specific information and does not imply recommendation or endorsement by the US Department of Agriculture. USDA is an equal opportunity provider and employer.

## Conflict of Interest Statement

B-gK is currently employed by Monsanto. VC and DD are employed by Bridgestone Americas. The remaining authors declare that the research was conducted in the absence of any commercial or financial relationships that could be construed as a potential conflict of interest. The work was supported by a Cooperative Research and Development Agreement (58-3k95-3-1653) between Bridgestone Americas, Incorporated and USDA-ARS Western Regional Research Laboratory. The funder played a role in the following: the study design, data analysis, and preparation of the manuscript.

## References

[B1] Allen S. G. Nakayama F. S. Dierig D. A. Rasnick B. A. (1987). Plant water relations, photosynthesis, and rubber content of young guayule plants during water stress. *Agron. J.* 79 1030–1035.

[B2] Archer B. L. Audley B. G. (1987). New aspects of rubber biosynthesis. *Bot. J. Linn. Soc.* 94 181–196. 10.1111/j.1095-8339.1987.tb01045.x

[B3] Arend M. Schnitzler J. P. Ehlting B. Hansch R. Lange T. Rennenberg H. (2009). Expression of the Arabidopsis mutant abi1 gene alters abscisic acid sensitivity, stomatal development and growth morphology in gray poplars. *Plant Physiol.* 151 2110–2119. 10.1104/pp.109.144956 19837818 PMC2785995

[B4] Backhaus R. A. Cornish K. Chen S. F. Huang D. S. Bess V. H. (1991). Purification and characterization of an abundant rubber particle protein from guayule. *Phytochemistry* 30 2493–2497. 10.1016/0031-9422(91)85088-h

[B5] Bae H. K. Kang H. G. Kim G. J. Eu H. J. Oh S. A. Song J. T. (2010). Transgenic rice plants carrying RNA interference constructs of AOS (allene oxide synthase) genes show severe male sterility. *Plant Breed.* 129 647–651. 10.1111/j.1439-0523.2010.01784.x

[B6] Biemelt S. Tschiersch H. Sonnewald U. (2004). Impact of altered gibberellin metabolism on biomass accumulation, lignin biosynthesis and photosynthesis in transgenic tobacco plants. *Plant Physiol.* 135 254–265. 10.1104/pp.103.036988 15122040 PMC429367

[B7] Brash A. R. (2009). Mechanistic aspects of CYP74 allene oxide synthases and related cytochrome P450 enzymes. *Phytochemistry* 70 1522–1531. 10.1016/j.phytochem.2009.08.005 19747698 PMC2783490

[B8] Cao X. Yan J. Lei J. Li J. Zhu J. Zhang H. (2017). De novo transcriptome sequencing of MeJA-induced *Taraxacum kok-saghyz* Rodin to identify genes related to rubber formation. *Sci. Rep.* 7 1–13. 10.1038/s41598-017-14890-z 29146946 PMC5691164

[B9] Chang Z. Li L. Pan Z. Wang X. (2008). Crystallization and preliminary X-ray analysis of allene oxide synthase, cytochrome P450 CYP74A2, from Parthenium argentatum. *Acta Crystallogr. Sec. F Struct. Biol. Cryst. Commun.* 64 668–670. 10.1107/S1744309108017545 18607105 PMC2443977

[B10] Christensen A. H. Quail P. H. (1996). Ubiquitin promoter-based vectors for high-level expression of selectable and/or screenable marker genes in monocotyledonous plants. *Transgenic Res.* 5 213–218. 10.1007/bf01969712 8673150

[B11] Cornish K. (2001). Similarities and differences in rubber biochemistry among plant species. *Phytochemistry* 57 1123–1134. 10.1016/s0031-9422(01)00097-8 11430985

[B12] Cornish K. Backhaus R. A. (1990). Rubber transferase activity in rubber particles of guayule. *Phytochemistry* 29 3809–3813. 10.1016/0031-9422(90)85337-f

[B13] Curtis O. F. Jr. (1947). Distribution of rubber and resins in guayule. *Plant Physiol.* 22 333–359. 10.1104/pp.22.4.333 16654108 PMC405879

[B14] Dai L. Nie Z. Kang G. Li Y. Zeng R. (2017). Identification and subcellular localization analysis of two rubber elongation factor isoforms on Hevea brasiliensis rubber particles. *Plant Physiol. Biochem.* 111 97–106. 10.1016/j.plaphy.2016.11.006 27915177

[B15] Dierig D. A. Ray D. T. Thompson A. E. (1989). Variation of agronomic characters among and between guayle lines. *Euphytica* 44 265–271. 10.1007/bf00037534

[B16] Dong N. Montanez B. Creelman R. A. Cornish K. (2006). Low light and low ammonium are key factors for guayule leaf tissue shoot organogenesis and transformation. *Plant Cell Rep.* 25 26–34. 10.1007/s00299-005-0024-2 16247613

[B17] Dong N. Ponciano G. McMahan C. M. Coffelt T. A. Johnson L. Creelman R. (2013). Overexpression of 3-hydroxy-3-methylglutaryl coenzyme A reductase in Parthenium argentatum (guayule). *Ind. Crops Prod.* 46 15–24. 10.1016/j.indcrop.2012.12.044

[B18] Downes E. W. Tonnet M. (1985). Effect of environmental conditions on growth and rubber production of guayule (Parthenium argentatum). *Aust. J. Agric. Res.* 36 285–294.

[B19] Garbarino J. E. Belknap W. R. (1994). Isolation of a ubiquitin-ribosomal protein gene (ubi3) from potato and expression of its promoter in transgenic plants. *Plant Mol. Biol.* 24 119–127. 10.1007/bf00040579 8111011

[B20] Gilliland M. G. van Staden J. (1986). Cyclic patterns of growth and rubber deposition in guayule *Parthenium argentatum*. Suggestions for a management programme. *S. Afr. J. Plant Soil* 3 21–26. 10.1080/02571862.1986.10634180

[B21] Gilliland M. G. van Staden J. Bruton A. G. (1984). Studies on the translocation system of guayule (Parthenium argentatum Gray). *Protoplasma* 122 169–177. 10.1007/bf01281694

[B22] Goss R. A. Benedict C. R. Keithly J. H. Nessler C. L. Stipanovic R. D. (1984). Cis- polyisoprene synthesis in guayule plants (Parthenium argentatum) exposed to low nonfreezing temperatures. *Plant Physiol.* 74 534–537. 10.1104/pp.74.3.534 16663456 PMC1066721

[B23] Hajdukiewicz P. Svab Z. Maliga P. (1994). The small, versatile pPZP family of Agrobacterium binary vectors for plant transformation. *Plant Mol. Biol.* 25 989–994. 10.1007/bf00014672 7919218

[B24] Hao B. Z. Wu J. L. (2000). Laticifer differentiation in *Hevea brasiliensis*: induction by exogenous jasmonic acid and linolenic acid. *Ann. Bot.* 85 37–43. 10.1006/anbo.1999.0995

[B25] Harms K. Atzorn R. Brash A. Kühn H. Wasternack C. Willmitzer L. (1995). Expression of a flax allene oxide synthase cDNA leads to increased endogenous jasmonic acid (JA) levels in transgenic potato plants but not to a corresponding activation of JA-responding genes. *Plant Cell* 7 1645–1654. 10.1105/tpc.7.10.1645 12242357 PMC161026

[B26] Heath R. L. Parker L. (1968). Photoperoxidation in isolated chloroplasts. I. Kinetics and stoichiometry of fatty acid peroxidation. *Arch. Biochem. Biophys.* 125 189–198. 10.1016/0003-9861(68)90654-1 5655425

[B27] Herrera-Estrella L. DeBlock M. Messens E. Hernalsteens J. P. Van Montagu M. Schell J. (1983). Chimeric genes as dominant selectable markers in plant cells. *EMBO J.* 2 987–996. 16453464 10.1002/j.1460-2075.1983.tb01532.x PMC555219

[B28] Hood E. E. Helmer G. L. Fraley R. T. Chilton M. (1986). The hypervirulence of *Agrobacterium tumefaciens* A281 is encoded a region of pTBo542 outside of T-DNA. *J. Bacteriol.* 168 1291–1301. 10.1128/jb.168.3.1291-1301.1986 3782037 PMC213636

[B29] Hou X. Ding L. Yu H. (2013). Crosstalk between GA and JA signaling mediates plant growth and defense. 32, 1067-1074. *Plant Cell Rep.* 32 1067–1074. 10.1007/s00299-013-1423-4 23525761

[B30] Hughes R. K. Belfield E. J. Ashton R. Fairhurst S. A. Göbel C. Stumpe M. (2006). Allene oxide synthase from Arabidopsis thaliana (CYP74A1) exhibits dual specificity that is regulated by monomer-micelle association. *FEBS Lett.* 580 4188–4194. 10.1016/j.febslet.2006.06.075 16831431

[B31] Hughes R. K. De Domenico S. Santino A. (2009). Plant cytochrome CYP74 family, Biochemical features, endocellular localization, activation mechanism in plant defense and improvements for industrial applications. *Chembiochem* 10 1122–1133. 10.1002/cbic.200800633 19322850

[B32] Illut D. C. Sanchez P. L. Coffelt T. A. Dyer J. M. Jenks M. A. Gore M. A. (2017). A century of guayule, comprehensive genetic characterization of the guayule (*Parthenium argentatum* A. Gray) USDA germplasm collection. *Ind. Crops Prod.* 109 300–309. 10.1016/j.indcrop.2017.08.029

[B33] Ji W. Benedict C. R. Foster M. A. (1993). Seasonal variations in rubber biosynthesis, 3- hydroxy-3-methylglutaryl-coenzyme A reductase, and rubber transferase activities in Parthenium argentatum in the Chihuahuan desert. *Plant Physiol.* 103 535–542. 10.1104/pp.103.2.535 12231959 PMC159013

[B34] Junglee S. Urban L. Sallanon H. Lopez-Lauri F. (2014). Optimized assay for hydrogen peroxide determination in plant tissue using potassium iodide. *Am. J. Analyt. Chem.* 5 730–736. 10.4236/ajac.2014.511081

[B35] Kajiura H. Suzuki N. Mouri H. Watanabe N. Nakazawa Y. (2018). Elucidation of rubber biosynthesis and accumulation in the rubber producing shrub, guayule (Parthenium argentatum Gray). *Planta* 247 513–526. 10.1007/s00425-017-2804-7 29116401

[B36] Kim J. S. Kim Y. O. Ryu H. J. Kwak Y. S. Lee J. Y. Kang H. (2003). Isolation of stress-related genes or rubber particles and latex in Fig Tree (Ficus carica) and their expressions by abiotic stress or plant hormone. *Plant Cell Physiol.* 44 412–414. 10.1093/pcp/pcg058 12721382

[B37] Konno K. (2011). Plant latex and other exudates as plant defense systems, roles of various defense chemical and proteins contained therein. *Phytochemistry* 52 1510–1530. 10.1016/j.phytochem.2011.02.016 21450319

[B38] Kuruvadi S. Canto D. J. Angulo-Sanchez J. L. (1997). Rubber content in different plant parts and tissues of Mexican guayule shrubs. *Ind. Crops Prod.* 7 19–25. 10.1016/s0926-6690(97)00033-2

[B39] Laudert D. Schaller F. Weiler E. W. (2000). Transgenic *Nicotiana tabacum* and *Arabidopsis thaliana* plants overexpressing allene oxide synthase. *Planta* 211 163–167. 10923718 10.1007/s004250000316

[B40] Lewinsohn T. M. (1991). The geographical distribution of plant latex. *Chemoecology* 2 64–68. 10.1007/bf01240668

[B41] Li L. Chang Z. Pan Z. Fu Z.-Q. Wang X. (2008). Modes of heme binding and substrate access for cytochrome P450 CYP74A revealed by crystal structures of allene oxide synthase. *Proc. Natl. Acad. Sci. U.S.A.* 105 13883–13888. 10.1073/pnas.0804099105 18787124 PMC2533679

[B42] Liu X. Li F. Tang J. Wang W. Zhang F. Wang G. (2012). Activation of the jasmonic acid pathway by depletion of the hydroperoxide lyase OsHPL3 reveals crosstalk between the HPL and AOS branches of the oxylipin pathway in rice. *PLoS One* 7:e50089. 10.1371/journal.pone.0050089 23209649 PMC3510209

[B43] Livak K. J. Schmittgen T. D. (2001). Analysis of relative gene expression data using real- time quantitative PCR and the 2-ΔΔCT Method. *Methods* 25 402–408. 10.1006/meth.2001.1262 11846609

[B44] Luttgeharm K. D. Chen M. Mehra A. Cahoon R. E. Markham J. E. Cahoon E. B. (2015). Overexpression of Arabidopsis ceramide synthases differentially affects growth, sphingolipid metabolism, programmed cell death and mycotoxin resistance. *Plant Physiol.* 169 1108–1117. 10.1104/pp.15.00987 26276842 PMC4587468

[B45] Macrae S. Gilliland M. G. Van Staden J. (1986). Rubber production in guayule, determination of rubber producing potential. *Plant Physiol.* 81 1027–1032. 10.1104/pp.81.4.1027 16664938 PMC1075480

[B46] Madhavan S. Greenblatt G. A. Foster M. A. Benedict C. R. (1989). Stimulation of isopentenyl pyrophosphate incorporation into polyisoprene in extracts from guayule plants (Parthenium argentatum Gray) by low temperature and 2- (3,4- dichloro- phenoxy)triethylamine. *Plant Physiol.* 89 506–511. 10.1104/pp.89.2.506 16666574 PMC1055872

[B47] Mei C. Qi M. Sheng G. Yang Y. (2006). Inducible overexpression of a rice oxide synthase gene increases the endogenous jasmonic acid level, PR gene expression, and host resistance to fungal infection. *Mol. Plant Microbe Interact.* 10 1127–1137. 10.1094/mpmi-19-1127 17022177

[B48] Mooibroek H. Cornish K. (2000). Alternative sources of natural rubber. *Appl. Microbiol. Biotechnol.* 53 355–365. 10.1007/s002530051627 10803889

[B49] Nawamawat K. Sakdapipanich J. T. Ho C. C. Ma Y. Song J. Vancso J. G. (2011). Surface nanostructure of Hevea brasiliensis natural rubber latex particles. *Colloids Surf. A* 390 157–166. 10.1016/j.saa.2011.07.024 21803643

[B50] Norton G. Pappusamy A. Yusof F. Pujade-Renaud V. Perkins M. Griffiths D. (2007). Characterization of recombinant *Hevea brasiliensis* allene oxide synthase, Effects of cyclooxygenase inhibitors, lipoxygenase inhibitors and salicylates on enzyme activity. *Plant Physiol. Biochem.* 45 129–138. 10.1016/j.plaphy.2007.01.003 17344058

[B51] Nyaka-Ngobisa A. I. C. Zainal-Abidin M. A. Wong M. Y. Wan-Noordin M. W. D. (2013). Neofusicoccum ribis associated with leaf blight on rubber (Hevea brasiliensis) in Peninsular Malaysia. *Plant Pathol. J.* 29 10–16. 10.5423/PPJ.OA.07.2012.0110 25288924 PMC4174792

[B52] Ohya N. Tanaka Y. Wititsuwannakul R. Koyama T. (2000). Activity of rubber transferase and rubber particle size in Hevea latex. *J. Rubb. Res.* 3 214–221.

[B53] Pan X. Welti R. Wang X. (2010). Quantitative analysis of major plant hormones in crude plant extracts by high-performance liquid chromatography-mass spectrometry. *Nat. Protoc.* 5 986–992. 10.1038/nprot.2010.37 20448544

[B54] Pan Z. Camara B. Gardner H. W. Backhaus R. A. (1998). Aspirin inhibition and acetylation of the plant cytochrome P450 allene oxide synthase, resembles that of animal prostaglandin endoperoxide H synthase. *J. Biol. Chem.* 273 18139–18145. 10.1074/jbc.273.29.18139 9660772

[B55] Pan Z. Durst F. Werck-Reichhart D. Gardner H. W. Camara B. Cornish K. (1995). The major protein of guayule rubber particles is a cytochrome P450. Characterization based on cDNA cloning and spectroscopic analysis of the solubilized enzyme and its reaction products. *J. Biol. Chem.* 270 8487–8494. 10.1074/jbc.270.15.8487 7721745

[B56] Park J. H. Halitschke R. Kim H. B. Baldwin I. T. Feldman K. A. Feyereisen R. (2002). A knock- out mutation in allene oxide synthase results in male sterility and defective wound signal transduction in Arabidopsis due to a block in jasmonic acid biosynthesis. *Plant J.* 31 1–12. 10.1046/j.1365-313x.2002.01328.x 12100478

[B57] Pearson C. H. Cornish K. Rath D. J. (2013). Extraction of natural rubber and resin from guayule using an accelerated solvent extractor. *Ind. Crops Prod.* 43 506–510. 10.1016/j.indcrop.2012.06.052

[B58] Pieterse C. M. J. Van der Does D. Zamioudis C. Leon-Reyes A. Van Wees S. C. M. (2012). Hormonal modulation of plant immunity. *Ann. Rev. Cell Dev. Biol.* 28 489–521. 10.1146/annurev-cellbio-092910-154055 22559264

[B59] Pirrello J. Leclercq J. Dessailly F. Rio M. Piyatrakul P. Kuswanhadi K. (2014). Transcriptional and post-transcriptional regulation of the jasmonate signaling pathway in response to abiotic and harvesting stress in *Hevea brasiliensis*. *BMC Plant Biol.* 14:341. 10.1186/s12870-014-0341-0 25443311 PMC4274682

[B60] Ponciano G. McMahan C. M. Xie W. Lazo G. R. Coffelt T. A. Collins-Silva J. (2012). Transcriptome and gene expression analysis in cold-acclimated guayule *(Parthenium argentatum*) rubber- producing tissue. *Phytochemistry* 79 57–66. 10.1016/j.phytochem.2012.04.007 22608127

[B61] Putter J. (1974). “Peroxidases,” in *Methods of Enzymatic Analysis* Vol. 2 ed. Bergmeyer H. U. (Cambridge, MA: Academic Press), 685–690.

[B62] Qi T. Wang J. Huang H. Liu B. Gao H. Liu Y. (2015). Regulation of jasmonate-induced leaf senescence by antagonism between bHLH subgroup IIIe and IIId factors in Arabidopsis. *Plant Cell* 27 1634–1649. 10.1105/tpc.15.00110 26071420 PMC4498205

[B63] Quan L. J. Zhang B. Shi W. W. Li H. Y. (2008). Hydrogen peroxide, a versatile molecule of the reactive oxygen species network. *J. Integr. Plant Biol.* 50 2–18. 10.1111/j.1744-7909.2007.00599.x 18666947

[B64] Radziah N. Z. Chee K. H. (1989). A new foliar disease of rubber. *Plant Pathol.* 38 293–296. 10.1007/s00484-018-1598-z 30136126

[B65] Ray D. T. Dierig D. A. Thompson A. E. Coffelt T. A. (1999). Registration of six guayule germplasms with high yielding ability. *Crop. Sci.* 39:300 10.2135/cropsci1999.0011183x003900010073x

[B66] Rockhold D. R. Chang S. Taylor N. Allen P. V. McCue K. F. Belknap W. R. (2008). Structure of two Solanum bulbocastanum polyubiquitin genes and expression of their promoters. *Am. J. Pot. Res.* 85 219–226. 10.1007/s12230-008-9015-5

[B67] Rojruthai P. Sakdapipanich J. T. Takahashi S. Hyegin L. Noike M. Koyama T. (2010). In vitro synthesis of high molecular weight rubber by Hevea small rubber particles. *J. Biosci. Bioeng.* 109 107–114. 10.1016/j.jbiosc.2009.08.009 20129092

[B68] Salvucci M. E. Barta C. Byers J. A. Canarini A. (2010). Photosynthesis and assimilate partitioning between carbohydrates and isoprenoid products in vegetatively active and dormant guayule, physiological and environmental constraints on rubber accumulation in a semiarid shrub. *Physiol. Plant.* 140 368–379. 10.1111/j.1399-3054.2010.01409.x 20727105

[B69] Sansatsadeekul J. Sakdapipanich J. Rojruthai P. (2011). Characterization of assocated proteins and phospholipids in natural rubber latex. *J. Biosci. Bioeng.* 111 628–634. 10.1016/j.jbiosc.2011.01.013 21354367

[B70] Savchenko T. Kolla V. A. Wang C.-Q. Nasafi Z. Hicks D. R. Phadungchod B. (2014). Functional convergence of oxylipin and abscisic acid pathways controls stomatal closure in response to drought. *Plant Physiol.* 164 1151–1160. 10.1104/pp.113.234310 24429214 PMC3938610

[B71] Schmidt T. Lenders M. Hillebrand A. van Deenen N. Munt O. Reichelt R. (2010). Characterization of rubber particles and rubber chain elongation in *Taraxacum kok-saghyz*. *BMC Biochem.* 11:11. 10.1186/1471-2091-11-11 20170509 PMC2836272

[B72] Siler D. J. Cornish K. (1994). Hypoallergenicity of guayule rubber particle proteins compared to Hevea latex proteins. *Ind. Crops Prod.* 2 307–313. 10.1016/0926-6690(94)90122-8

[B73] Song S. Qi T. Wasternack C. Xie D. (2014). Jasmonate signaling and crosstalk with gibberellin and ethylene. *Curr. Opin. Plant Biol.* 21 112–119. 10.1016/j.pbi.2014.07.005 25064075

[B74] Song W. C. Brash A. R. (1991). Purification and characterization of an allene oxide synthase and identification of the enzyme as a cytochrome P450. *Science* 253 781–784. 10.1126/science.1876834 1876834

[B75] Song W. C. Funk C. D. Brash A. R. (1993). Molecular cloning of an allene oxide synthase, a cytochrome P450 specialized for the metabolism of fatty acid hydroperoxides. *Proc. Natl. Acad. Sci. U.S.A.* 90 8519–8523. 10.1073/pnas.90.18.8519 8378325 PMC47388

[B76] Stenzel I. Hause B. Maucher H. Pitzschke A. Miersch O. Ziegler J. (2003). Allene oxide cyclase dependence of the wound response and vascular bundle-specific generatin of jasmonates in tomato-amplication in wound signalling. *Plant J.* 33 577–589. 10.1046/j.1365-313x.2003.01647.x 12581315

[B77] Sundar D. Chaitanya K. V. Jutur P. P. Reddy R. A. (2004). Low temperature-induced changes in antioxidative metabolism in rubber-producing shrub, guayule (Parthenium argentatum Gray). *Plant Growth Regul.* 44 175–181. 10.1023/b 11099962

[B78] Sundar D. Reddy R. (2000). Low night temperature-induced changes in photosynthesis and rubber accumulation in guayule (Parthenium argentatum Gray). *Photosynthetica* 3 421–427.

[B79] Tan D. Hu X. Fu L. Kumpeangkeaw A. Ding Z. Sun X. (2017). Comparative morphology and transcriptome analysis reveals distinct functions of the primary and secondary laticifer cells in the rubber tree. *Sci. Rep.* 7 1–17. 10.1038/s41598-017-03083-3 28600566 PMC5466658

[B80] Tangpakdee J. A. Tanaka Y. (1998). Why rubber trees produce polysioprene-a possible role of natural rubber in the Hevea Tree. *J. Rubb. Res.* 1 77–83.

[B81] Tanimoto E. (2012). Tall or short? Slender or thick? A plant strategy for regulating elongation growth of roots by low concentrations of gibberellin. *Ann. Bot.* 110 373–381. 10.1093/aob/mcs049 22437663 PMC3394641

[B82] Turan S. Kumar S. Cornish K. (2014). Photosynthetic response of in vitro guayule plants in low and high lights and the role of non-photochemical quenching in plant acclimation. *Ind. Crops Prod.* 54 266–271. 10.1016/j.indcrop.2014.01.022

[B83] Valdes Franco J. A. Wang Y. Huo N. Ponciano G. Colvin H. A. McMahan C. M. (2018). Modular assembly of transposable element arrays by microsatellite targeting in the guayule and rice genomes. *BMC Genomics* 19:271. 10.1186/s12864-018-4653-6 29673330 PMC5907723

[B84] van Beilen J. B. Poirier Y. (2007). Establishment of new crops for the production of natural rubber. *Trends Biotechnol.* 25 522–529. 10.1016/j.tibtech.2007.08.009 17936926

[B85] von Malek B. van der Graaff E. Schneitz K. Keller B. (2002). The Arabidopsis male- sterile mutant dde2-2 is defective in the ALLENE OXIDE SYNTHASE gene encoding one of the key enzymes of the jasmonic acid biosynthesis pathway. *Planta* 216 187–192. 10.1007/s00425-002-0906-2 12430030

[B86] Walters D. Heil M. (2007). Costs and trade-offs associated with induced resistance. *Physiol. Mol. Plant Pathol.* 71 3–17. 10.1016/j.pmpp.2007.09.008

[B87] Wang C. Avdiushko S. Hildebrand D. F. (1999). Overexpression of a cytoplasm- localized allene oxide synthase promotes the wound-induced accumulation of jasmoinic acid in transgenic tobacco. *Plant Mol. Biol.* 40 783–793. 10487213 10.1023/a:1006253927431

[B88] Wasternack C. Hause B. (2013). Jasmonates, biosynthesis, perception, signal transduction and action in plant stress response, growth and development. An update to the 2007 review in Annals of Botany. *Ann. Bot.* 111 1021–1058. 10.1093/aob/mct067 23558912 PMC3662512

[B89] Wasternack C. Song S. (2017). Jasmonates, biosynthesis, metabolism, and signaling by proteins activating and repressing transcription. *J. Exp. Bot.* 68 1303–1321. 10.1093/jxb/erw443 27940470

[B90] Whalen M. McMahan C. Shintani D. (2013). “Development of crops to produce industrially useful natural rubber,” in *Isoprenoid Synthesis in Plants and Microorganisms, New Concepts and Experimental Approaches*, eds Bach T. Rohmer M. (New York, NY: Springer), 329–345. 10.1007/978-1-4614-4063-5_23

[B91] Yamashita S. Mizuno M. Hayashi H. Yamaguchi H. Miyagi-Inoue Y. Fushihara K. (2017). Purification and characterization of small and large rubber particles from *Hevea brasiliensis*. *Biosci. Biotechnol. Biochem.* 82 1011–1020. 10.1080/09168451.2017.1401913 29191089

[B92] Yao C. Finlayson S. A. (2015). Abscisic acid is a general negative regulator of Arabidopsis axillary bud growth. *Plant Physiol.* 169 611–626. 10.1104/pp.15.00682 26149576 PMC4577412

[B93] Yu J. H. Zeng X. Yang S. G. Huang H. S. Tian W. M. (2008). Relationship between rate of laticifer differentiation, number of laticifer rows and rubber yield among 1981 IRRDB Germplasm collections of Hevea brasiliensis. *J. Rubb. Res.* 11 43–51.

[B94] Zhu X. Chen J. Xie Z. Gao J. Ren G. Gao S. (2015). Jasmonic acid promotes degreening via MYC2/3/4- and ANAC019/055/072-mediatd regulation of major chlorophyll catabolic genes. *Plant J.* 84 597–610. 10.1111/tpj.13030 26407000

