# Supplementary material for: Downregulation of a CYP74 Rubber Particle Protein Increases Natural Rubber Production in Parthenium argentatum
Source: Front Plant Sci. 2019 Jun 26;10:760. doi: 10.3389/fpls.2019.00760 (PMC6607968; doi:10.3389/fpls.2019.00760)
Supplement: Supplementary file 1 [file Data_Sheet_1.pdf]

Supplementary Material

Downregulation of a CYP74 rubber particle protein increases natural rubber production in *Parthenium argentatum*

Dante F. Placido, Niu Dong, Chen Dong, Von Mark V. Cruz, David A. Dierig, Rebecca E. Cahoon, Byung-guk Kang, Trinh Huynh, Maureen Whalen, Grisel Ponciano[*] and Colleen McMahan[1]*

***Corresponding authors**

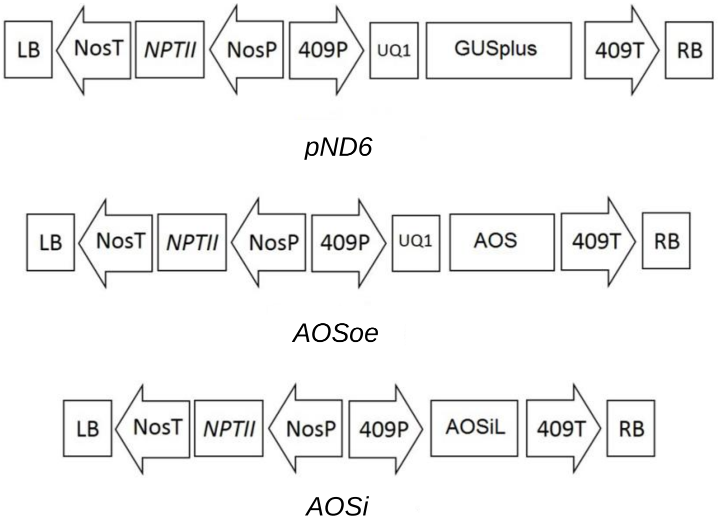

Supplementary Figure S1. T-DNA region of transformation constructs. *pND6*, *β-glucuronidase* (*GUSplus*) reporter gene driven by potato *ubiquitin 1* (UQ1) and 409 promoter (*409P*) with potato 409 terminator (*409T*); *AOSoe*, the *GUSplus* gene in *pND6* is replaced with *Parthenium argentatum AOS* gene; *AOSi*, the *GUSplus* gene in *pND6* is replaced with a hairpin RNA construct built with an inverted repeat of partial *P. argentatum AOS* with bialaphos resistance (*BAR*) gene insert between the inverted repeats. All constructs have the kanamycin resistance (*nptII*) gene driven by the *nopaline synthase* gene promoter (*NosP*) with *Nos* gene terminator (*NosT*); LB, left border; RB, right border.

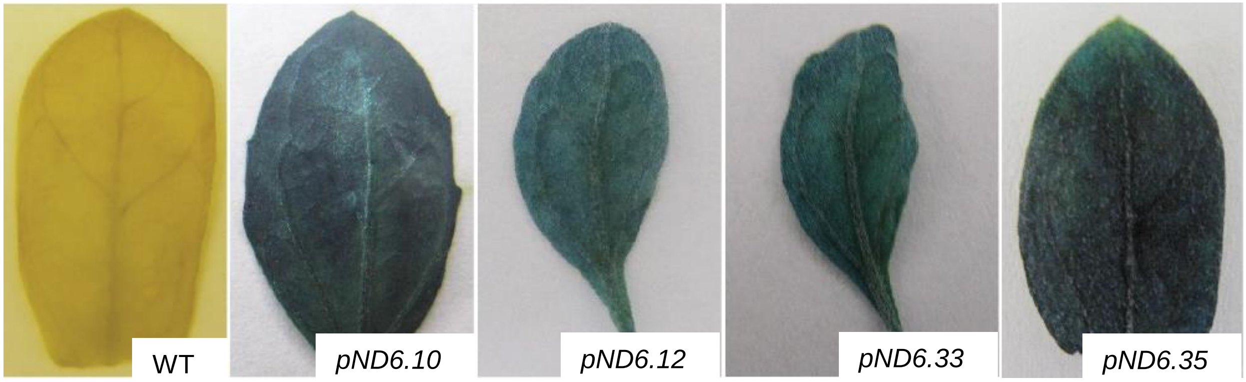

Supplementary Figure S2. β-glucuronidase histochemical assay for *pND6* transgenic guayule plants. The expression of the *GUSplus* gene in leaves of guayule plants was confirmed by histochemical staining (blue stained leaves). *GUSplus* expression was not detected in non-transformed control plant tissues, WT.

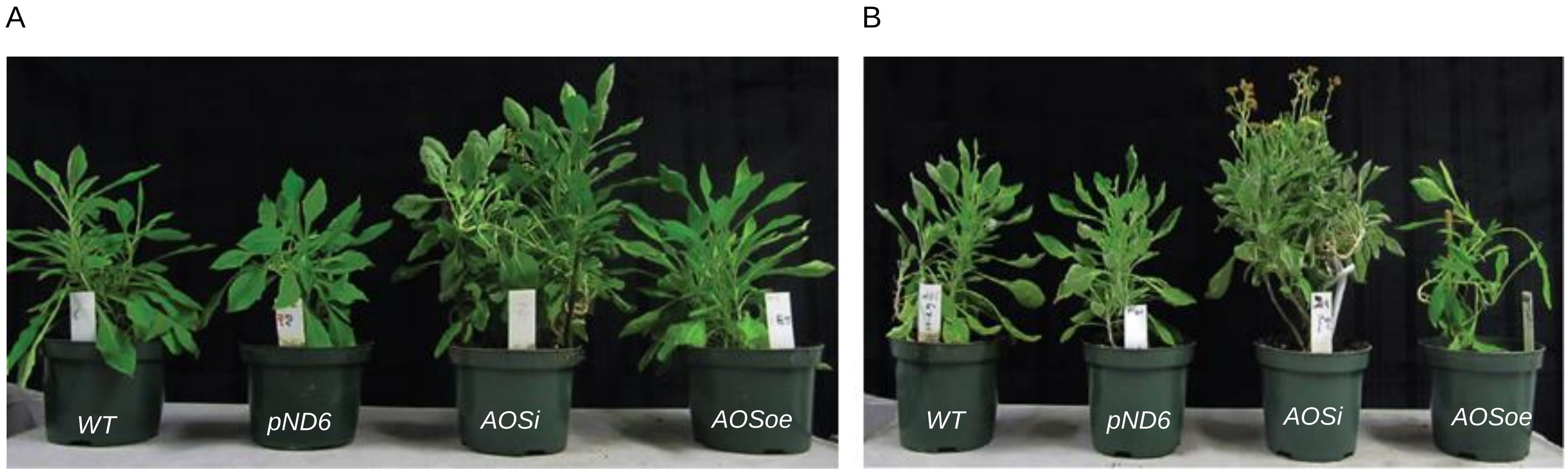

Supplementary Figure S3. Comparison of representative 5-month-old guayule WT and *AOS* transgenic lines grown in growth chamber conditions at A) room temperature and B) cold temperature.

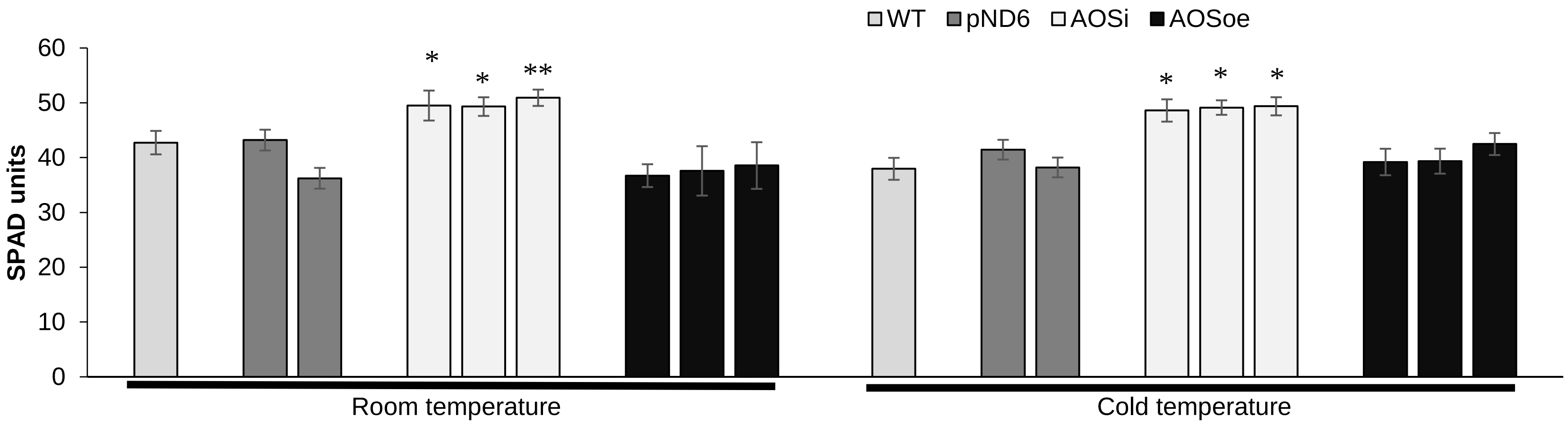

Supplementary Figure S4. Leaf chlorophyll concentration measurements from middle leaf using SPAD-502 chlorophyll meter. (*) and (**) indicates significant difference in comparison to G7-11 at $p > 0.05$ and 0.005, respectively.

A

B

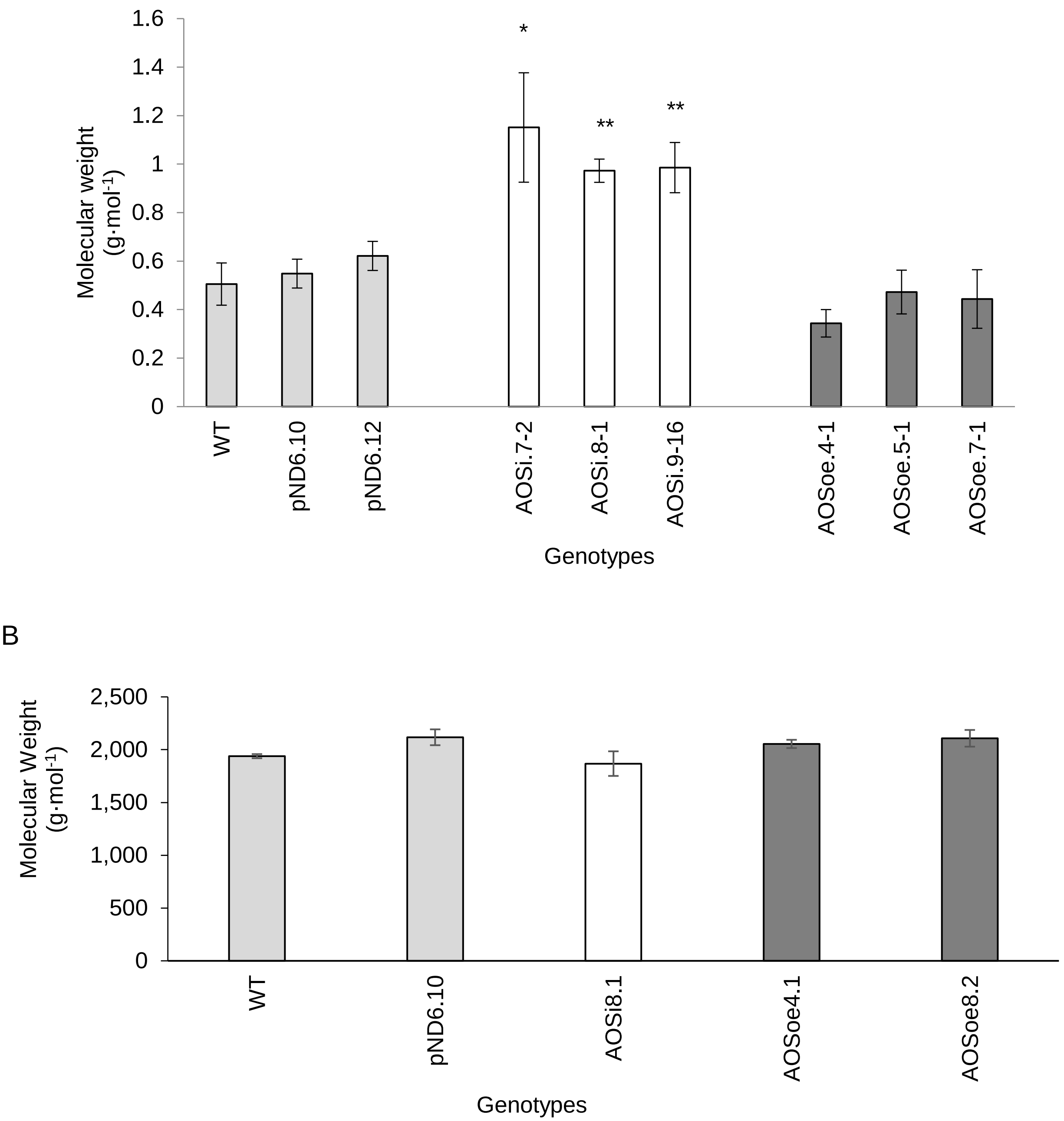

Supplementary Figure S5. Rubber molecular weight determination by gel permeation chromatography. A) Tissue culture grown plants. Rubber extracted by ASE from 8-week-old plants. B) Greenhouse grown plants. Rubber particles extracted from the stembark tissue of one-year old plants. The * and ** indicate significant difference in comparison to the WT at $p > 0.05$ and $p > 0.005$, respectively. Error bars represent SD from the average of three biological replicates.

A

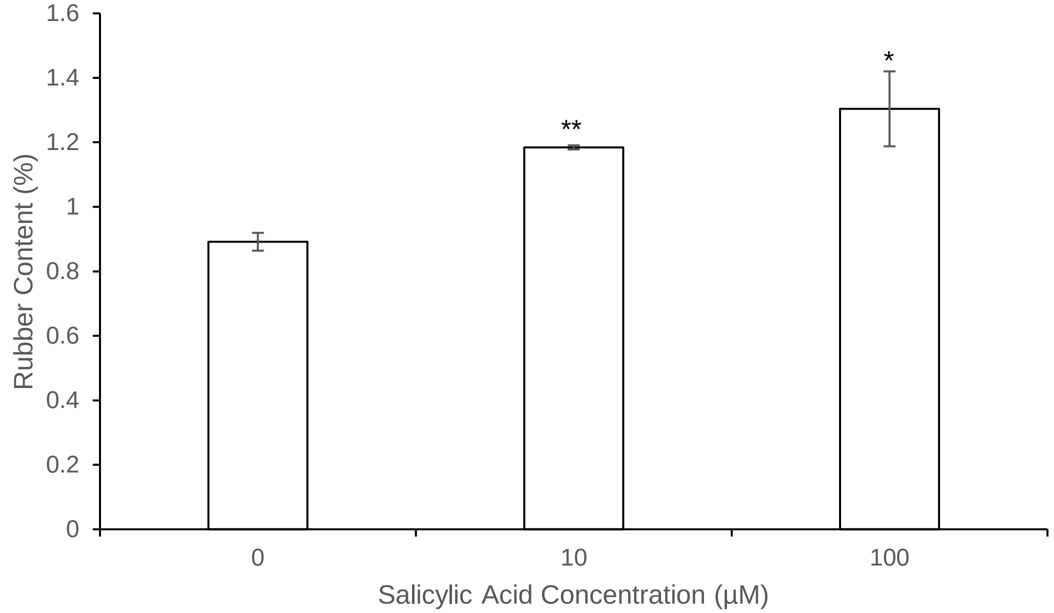

B

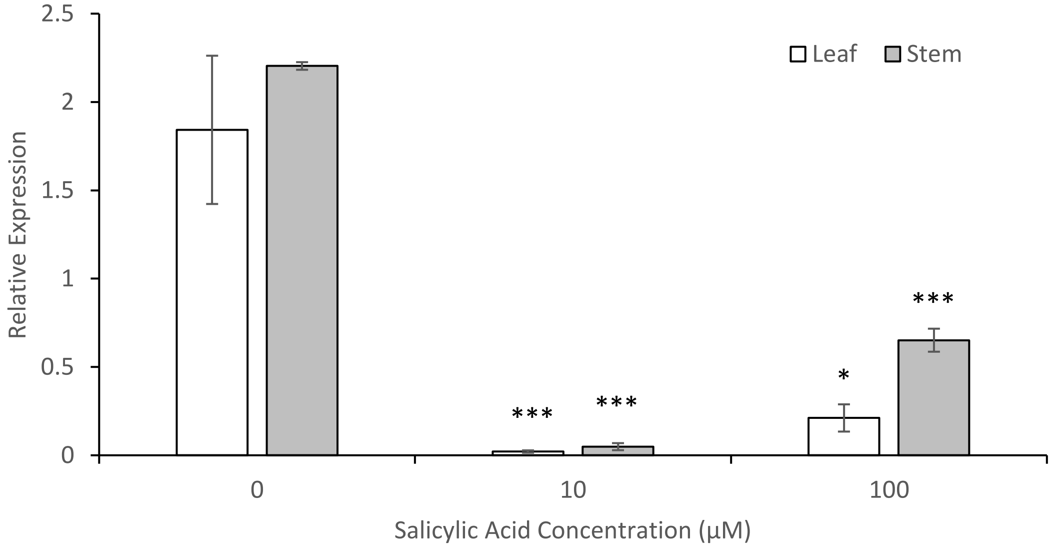

Supplementary Figure S6. Effect of salicylic acid on rubber content and gene expression in 8-week-old wild-type G7-11 guayule cultivar grown in tissue culture. A) Rubber content extracted by ASE; B) *AOS* relative expression normalized to the *18S* reference gene. Error bars represent SD from the average of three biological replicates from each experiment. (*), (**) and (***) indicate significant difference in comparison to WT at $p > 0.05$, 0.005 and 0.0005, respectively. Data are representative of three independent experiments.

```
PaAOS          ATGGACCCATCGTCTAAA---CCCCTCCGTGAAATCCCCGGCTCTTATGGCATTCCTTTC
PaAOSL4        ------------------------------------------------------------
PaASOL3        ATGGATCCATCATCTGAAGCCCCTCCTCGTGAAATCCCTGGTTCCTACGGCATTCCTTTT
PaAOSL2        ------------------------------------------------------------

PaAOS          TTTCAACCGATAAAAGACCGATTGGAGTATTTTTACGGGACCGGAGGTCGAGACGAGTAC
PaAOSL4        ------------------------------------------------------------
PaASOL3        ATCCAACCGATCAAAGACCGGTTAGAGTATTTTTACGGGACCGGAGGCCCAGATGGGTTC
PaAOSL2        ------------------------------------------------------------

PaAOS          TTCCGGTCCCGCATGCAAAAATACCAATCCACGGTATTTCGAGCCAACATGCCACCGGGC
PaAOSL4        ------------------------------------------------------------
PaASOL3        TTCCAGTCCCGCGTTCAAAAATACCAATCCACTGTGTTCCGTACCAATATGCCACCCGGC
PaAOSL2        ------------------------------------------------ATGCCACCTGGC

PaAOS          CCTTTCGTAAGCAGCAACCCGAAGGTCATCGTCCTACTCGACGCCAAAAGCTTTCCGATA
PaAOSL4        ------------------------------------------------------------
PaASOL3        CCTTTTATAAGCAGCAACCCAAAAGTCATTGTCCTCTTAGACGCCAAAAGCTTTCCCGTA
PaAOSL2        CCATTCATCTCCAAGAATCCAAACGTCGTCGTTTTACTCGACGCCAAAAGCTTCCCAACC

PaAOS          CTCTTTGATGTATCCAAAGTCGAGAAGAAAGATTTGTTCACCGGAACTTACATGCCGTCA
PaAOSL4        ------------------------------------------------------------
PaASOL3        TTGTTTGATGTTTCTAAAGTCGAGAAGAAAGATTTATTTACCGGAACTTATATGCCGTCA
PaAOSL2        CTTTTCGATGTCTCTAAAGTCGAAAAGAAAGACTTATTCACCGGCACTTACATGCCCTCC

PaAOS          ACCAAACTCACTGGCGGCTACCGCGTACTCTCGTACCTCGACCCATCCGAACCTAGACAT
PaAOSL4        ------------------------------------------------------------
PaASOL3        ACTAACCTCACTGGCGGCTACCGCGTACTCTCGTACCTCGACCCATCCGAACCTAGACAT
PaAOSL2        ACTGAACTCACCGGCGGCCACCGTGTCCTCTCTTATCTTGACCCTTCTGAGCCCAAACAC

PaAOS          GCTCAACTTAAGAACCTCTTGTTCTTCATGCTTAAAAATTCAAGCAACCGAGTCATTCCT
PaAOSL4        ------------------------------------------------------------
PaASOL3        GCTCAACTTAAGAACCTCTTGTTCTTCATGCTTAAAAATTCAAGCAACCGAGTCATTCCT
PaAOSL2        GGCCCATTAAAAAACATGGTCTTCTTCATGCTCAAATCATCCATATCAAGAGTCTTGCCC

PaAOS          CAGTTCGAAACCACTTACACCGAACTCTTTGAAGGTCTTGAAGCCGAGCTAGCCAAAAAC
PaAOSL4        ------------------------------------------------------------
PaASOL3        CAGTTCCAAACAACTTACACCGAACTCTTTGAAGGTCTTGAAACCGAATTGGCCAAAAAT
PaAOSL2        GAATTCGAAACCACTTATACCGAACTTTTTGACGGGCTTGAATCCGAGCTGGCCCAAAAG

PaAOS          GGGAAAGCCGCGTTCAACGATGTTGGTGAACAAGCGGCTTTCCGGTTTTTGGGCAGGGCT
PaAOSL4        ------------------------------------------------------------
PaASOL3        GGGAAAGCCGCGTTCAACGACGTTGGTGAACAAGCGGCTTTCCGGTTTTTGGGCCGGGCT
PaAOSL2        GGTAAAGCTCTTTTTAACGATGTCGGCGAACAGGCCGCGTTTCGGTTTCTAGGCCGGGCG

PaAOS          TATTTTAACTCGAACCCGGAAGAAACCAAACTAGGAACTAGTGCGCCTACGTTAATTAGC
PaAOSL4        ------------------------------------------------------------
PaASOL3        TATTTCAACTCTAACCCGGAAGAAACCAAACTAGGAACTAGTGCGCCGAAGTTAATTACC
PaAOSL2        TATTTAGAAACCAACCCGGAAGAAACCAAGATTGGAAAAGACGGCCCGAAGTTAATTAGC
```

```
PaAOS           TCGTGGGTGTTATTTAATCTTGCCCCCACGCTCGACCTCGGACTTCCGTGGTTCTTGCAG
PaAOSL4         ------------------------------------------------------------
PaASOL3         ACGTGGGTGTTGTTCAATCTTAGCCCGATAGGCACTGCTGGACTTCCGTGGTTCTTGGAG
PaAOSL2         ACGTGGGTGTTATTTAACCTCGGGCCGTTACTCCGTCTTGGACTTCCTTGGTTCGTGGAG

PaAOS           GAACCTCTTCTACACACTTTCCGACTGCCGGCGTTCCTGATTAAGAGTACTTACAACAAA
PaAOSL4         ------------------------------------------------------------
PaASOL3         GACCCTCTTATCCACACTTTCCGACTGCCGTCGTTTCTGGTAAAGAGTAACTACAACAAG
PaAOSL2         GAGCCGCTCCTTCACACTTTTCGACTCCCTCCCGCGCTTGTAAAGAAAAATTATAACAAA

PaAOS           CTTTACGATTATTTCCAGTCGGTTGCGACTCCGGTTATGGAACAAGCAGAAAAATTAGGG
PaAOSL4         ------------------------------------------------------------
PaASOL3         CTTTACGATTATTTTGAGTCGGCTGCAACTCAGGTTGTGGAGCAAGCAGAAATATTAGGG
PaAOSL2         CTTTATGAGTTTTTTGAATCGTTTTCGGGTCCGATTATTGAACATGCCGAATCGTTGGGG

PaAOS           GTTCCGAAGGATGAAGCTGTGCACAATATCTTATTCGCGGTTTGCTTCAATACTTTTGGT
PaAOSL4         ------------------------------------------------------------
PaASOL3         GTTCCGAAAGATGAAGCTTTGCACAATATCTTATTCGCGGTTTGCTTCAATACTTTTGGT
PaAOSL2         ATTTCCAAAGAAGAAGCGGTTCACAATATTTTGTTTACAATTTGTTTTAATACGTTTGGC

PaAOS           GGTGTTAAGATCCTCTTCCCGAATACACTCAAATGGATCGGACTTGCTGGTGAGAATTTG
PaAOSL4         ------------------------------------------------------------
PaASOL3         GGGATGAAGATCCTCTTCCCTAATACACTCAAATGGATCGCACTTGCTGGTGAGAATTGG
PaAOSL2         GGGATCAAGATTTTGTTTCCGAATACGCTTAAATGGATCGGTCGGGCTGGGACGAATTTG

PaAOS           CATACCCAATTGGCGGAAGAGATTAGAGGTGCTATAAAATCATACGGGGACGGTAACGTG
PaAOSL4         ------------------------------------------------------------
PaASOL3         CATAACCAACTGGCGAAAGAGATCAGAGGTGCTATAGAATCATACGGTGGCGGTAAGGTG
PaAOSL2         CATACCCAATTGGCGGAAGAGATTCGGGGCGCGATTAAGGATCACGG---CGGGAAGGTG

PaAOS           ACGCTGGAAGCAATCGAGCAGATGCCGTTGACGAAGTCAGTGGTGTACGAGTCCCTCAGG
PaAOSL4         ---------------------ATGCCGTTGACGAAGTCAGTGGTGTACGAGTCCCTCAGG
PaASOL3         ACGCTGGAAGCGATCGAGCAGATGCCATTGATGAAGTCGGTTGTGTACGAGTCCCTCAGG
PaAOSL2         ACGATGGCGGCGATGGAGCAGATGCCGTTGATGAAATCCGTCGTGTACGAGTCACTCAGG
                                     ***** **** *** ** ** *********** ******

PaAOS           ATTGAACCACCAGTGCCTCCGCAATATGGAAAAGCCAAAAGCAACTTTACCATAGAGTCA
PaAOSL4         ATTGAACCACCAGTGCCTCCGCAATATGGAAAAGCCAAAAGCAACTTTACCATAGAGTCA
PaASOL3         ATTGATCCACCAGTACCACCTCAATATGGAAAAGCCAAAAGCAACTTTACTATTCAGTCA
PaAOSL2         ATCGAACCCCCTGTGGCATTGCAATACGGGAAAGCTAAACGCGACTTGACAATCGAGTCA
                ** ** ** ** **  *    ***** ** ***** *** ** **** ** **  *****

PaAOS           CACGACGCCACTTTCGAAGTCAAAAAAGGAGAAATGTTATTCGGGTACCAACCGTTTGCA
PaAOSL4         CACGACGCCACTTTCGAAGTCAAAAAAGGAGAAATGTTATTCGGGTACCAACCGTTTGCA
PaASOL3         CATGATGCCACTTTTGAAATCAAAAAAGGTGAAATGTTATTCGGATACCAACCGTTTGCA
PaAOSL2         CACGACGCTGTTTTCAAAGTCAAAGAAGGGGAAATGTTGTTCGGGTACCAACCATTTGCG
                ** ** **   ***  ** ***** **** ******** ***** ******** *****

PaAOS           ACCAAGGACCCAAAAGTATTTGACCGACCTGAGGAATATGTCCCTGATCGGTTCGTTGGG
PaAOSL4         ACCAAGGACCCAAAAGTATTTGACCGACCTGAGGAATATGTCCCTGATCGGTTCGTTGGG
PaASOL3         ACCAAGGACCCGAAAGTATTTGACCGACCTGAGGAGTTTGTCCCTGATCGGTTTGTTGGG
PaAOSL2         ACCAAGGATCCGAAAGTGTTTGATCGACCCGAAGAATTTGTTCCGGATCGCTTTGTGGGA
                ******** ** ***** ***** ***** ** ** * *** ** ***** ** ** **

PaAOS           GATGGCGAGGCATTGTTGAAGTACGTATGGTGGTCTAATGGGCCGGAGACAGAGAGTCCG
PaAOSL4         GATGGCGAGGCATTGTTGAAGTACGTATGGTGGTCTAATGGGCCGGAGACAGAGAGTCCG
PaASOL3         GACGGGGAGGCGTTGTTGAAGTATGTTTTGTGGTCTAATGGGCCGGAGACAGAGAGCCCG
PaAOSL2         GCGGGTGAGGAGCTTTTGAAGTATGTGACATGGTCTAACGGACCGGAGACGGAGGGGCCC
                *  ** ****   * ******** **    ******** ** ******** *** * **
```

```
PaAOS       ACAGTTGAAAATAAACAATGTGCCGGAAAAGACTTTGTCGTGCTTATAACGAGGTTGTTT
PaAOSL4     ACAGTTGAAAATAAACAATGTGCCGGAAAAGACTTTGTCGTGCTTATAACGAGGTTGTTT
PaASOL3     ACCATTGGAAATAAGCAATGTGCCGGGAAAAACTTTGTCGTGCTGATAACGAGGTTGTTT
PaAOSL2     ACAGTGGCGAACAAGCAATGTGCCGGAAAAGACTTTGTGGTGTTGATAACTCGGTTGTTT
            **  * *  ** ** *********** *** ******* *** * *****  ********

PaAOS       GTCATTGAACTTTTCCGGCGATATGACTCTTTTGAAATCGAATTAGGCGAGTCTCCTTTG
PaAOSL4     GTCATTGAACTTTTCCGGCGATATGACTCTTTTGAAATCGAATTAGGCGAGTCTCCTTTG
PaASOL3     GTCATTGAATTTTTCCGTCGATACGACTCGTTTGAAGTGGAGTCAAGCGCTTCTGCTTTG
PaAOSL2     GTTGTTGAACTTTTTCGGCGGTATGATTCGTTTGATATTGAAGTCGCTGCGTCTCCGTTA
            **  ***** **** ** ** ** ** ** *****  * **       *  *** * **

PaAOS       GGTGCAGCTGTCACACTTACGTCCCTGAAGAGAGCTAGTATATGA--
PaAOSL4     GGTGCAGCTGTCACACTTACGTCCCTGAAGAGAGCTAGTATATGATT
PaASOL3     GGTGCGGGAATTACATTTACGTCCCTGAAGAGGGCCAGTATATGA--
PaAOSL2     GGTGCTAAAGTTACGTTAACTTCTCTCAAGAGAGCTCGTGCGTGA--
            *****     * **  * ** ** ** ***** **  **   ***
```

Supplementary Figure S7. Nucleotide sequence alignment of guayule AOS cDNA (PaAOS, X78166) with guayule stembark RNASeq sequences (PaAOSL2-4, AOS-like sequences 2-4). Red font corresponds to AOS RNAi target region. Alignment executed with Clustal 2.1.

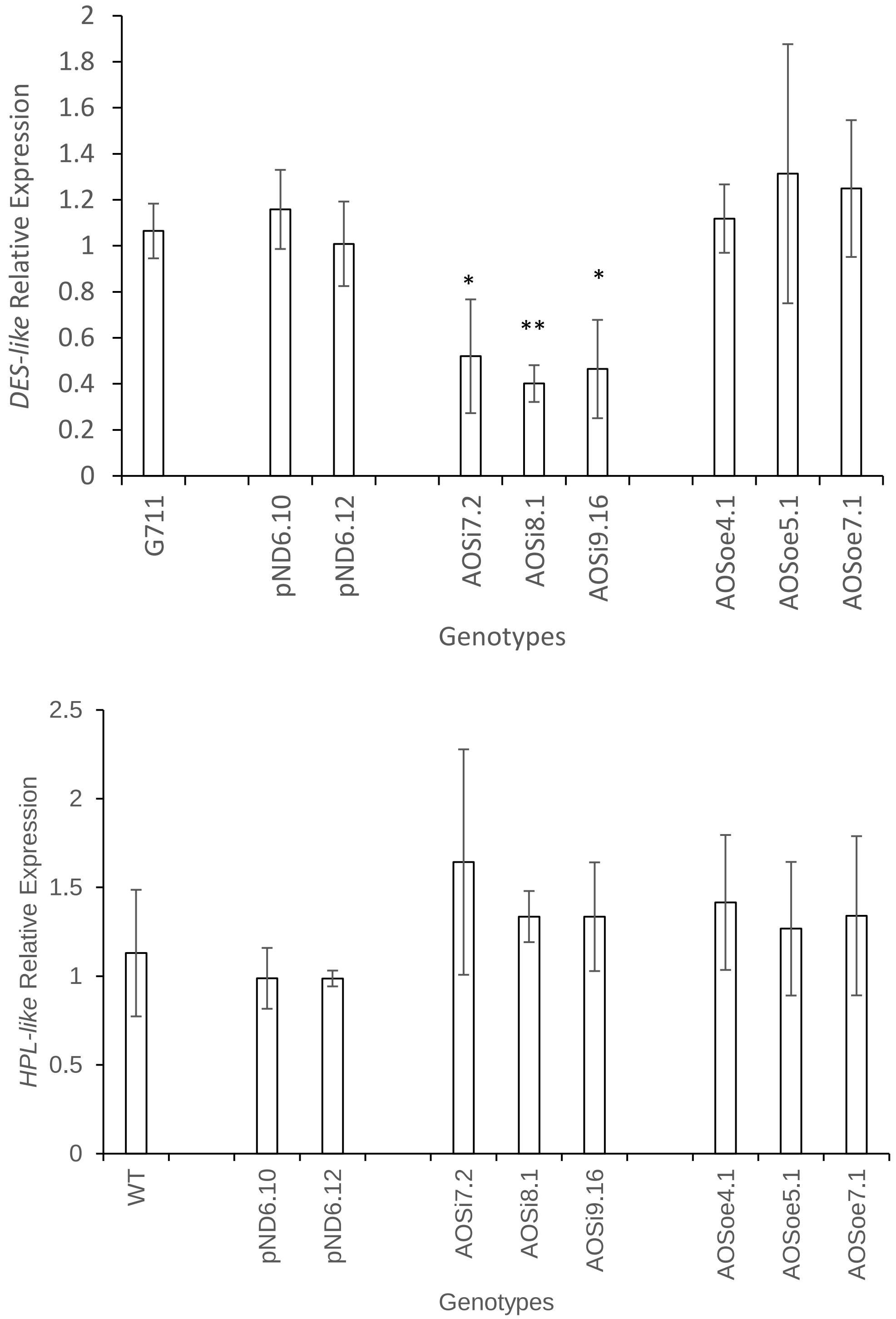

Supplementary Figure S8. Expression analysis of putative *DES* and *HPL* in guayule. WT, wild-type *G7-11*; *pND6*, vector control; *AOSi*, downregulated *AOS*; *AOSoe*, overexpressed *AOS*. Asterisks * and ** indicate significant difference in comparison to WT at p > 0.05 and p > 0.005 respectively. Total RNA from stembark of 5-month-old growth chamber-grown control and transgenic lines were template for qPCR. Expression levels were compared to WT and normalized to the *18S* reference gene. Average expression is from three biological replicates ± SD.

Supplementary Table S1. Rubber content of 8-week-old tissue culture-grown (leaf and stem) guayule control and transgenic lines by Accelerated Solvent Extraction (ASE). The percent rubber content is the average dry weight of three biological replicates ± SD. The asterisks (*), (**) and (***) indicate significant difference in comparison to WT at p > 0.05, 0.005 and 0.0005, respectively.

P. argentatum Genotypes	Average Rubber Content (%)
WT 1	1.01 ± .01
WT 2	1.11 ± .02
pND6 12	1.13 ± 0.2
pND6 33	1.10 ± 0.1
pND6 35	1.04 ± 0.2
AOSi 5.1	1.8 ± 0.1*
AOSi 7.2	2.0 ± 0.3**
AOSi 8.1	2.1 ± 0.04***
AOSi 8.2	1.7 ± 0.1**
AOSi 9.15	1.7 ± 0.02**
AOSi 9.16	2.3 ± 0.4*
AOSi 12.1	2.46 ± 0.3*
AOSi 12.3	1.62 ± 0.002***
AOSoe 4.1	0.96 ± 0.2
AOSoe 4.2	0.85 ± 0.1
AOSoe 5.1	1.09 ± 0.1
AOSoe 5.2	1.23 ± 0.1
AOSoe 7.1	0.96 ± .02
AOSoe 8.2	1.23 ± 0.1
AOSoe 11.5	1.23 ± 0.1
